# Synthetic community with six *Pseudomonas* strains screened from garlic rhizosphere microbiome promotes plant growth

**DOI:** 10.1111/1751-7915.13640

**Published:** 2020-08-06

**Authors:** Lubo Zhuang, Yan Li, Zhenshuo Wang, Yue Yu, Nan Zhang, Chang Yang, Qingchao Zeng, Qi Wang

**Affiliations:** ^1^ Department of Plant Pathology MOA Key Lab of Pest Monitoring and Green Management College of Plant Protection China Agricultural University Beijing 100193 China

## Abstract

The rhizosphere microbiome plays an important role in the growth and health of many plants, particularly for plant growth‐promoting rhizobacteria (PGPR). Although the use of PGPR could improve plant production, real‐world applications are still held back by low‐efficiency methods of finding and using PGPR. In this study, the structure of bacterial and fungal rhizosphere communities of Jinxiang garlic under different growth periods (resume growth, bolting and maturation), soil types (loam, sandy loam and sandy soil) and agricultural practices (with and without microbial products) were explored by using amplicon sequencing. High‐efficiency top‐down approaches based on high‐throughput technology and synthetic community (SynCom) approaches were used to find PGPR in garlic rhizosphere and improve plant production. Our findings indicated that *Pseudomonas* was a key PGPR in the rhizosphere of garlic. Furthermore, SynCom with six *Pseudomonas* strains isolated from the garlic rhizosphere were constructed, which showed that they have the ability to promote plant growth.

## Graphical abstract

This manuscript focused on the construction of a synthetic community with *Pseudomonas* strains isolated from the garlic rhizosphere and that this synthetic community have the ability to promote plant growth.

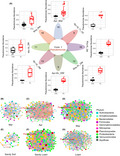

## Introduction

Plants assemble microbiomes from soil by a multistep model, which might be generally applied to land plants (Edwards *et al*., 2015; van der Heijden and Schlaeppi, 2015). Rhizosphere plays a significant role in the assembly process of microbes and the interaction between plants and microbes. The assembly process of microbes is influenced by the plant root, which shapes the rhizosphere microbiome into a stable structure. Plant growth‐promoting rhizobacteria (PGPR) are rhizosphere bacteria that have positive effects on the growth of plants and are often enriched in the rhizosphere compared with bulk soil (Lugtenberg and Kamilova, 2009). PGPR enhance crop yield by assisting the nutrient absorption of plants from soil (Bolan, 1991; Zhang *et al*., 2009) and producing plant growth‐promoting substances, such as hormone auxin (van Loon, 2007). Under infections of plant diseases, PGPR are recruited into the rhizosphere environment by plants to help with the suppression of pathogens (Berendsen *et al*., 2012). The structure of rhizosphere microbiomes is affected by the assembly process and plant growing periods. For example, as rice grows, the root recruits bacteria such as *Nitrospira*, which modulates the nitrogen cycle and promotes crop growth (Zhang *et al*., 2018). In addition, fertile soil types and good agricultural practices, such as artificially applying beneficial microbial products, can increase crop yields. Different soil types possess different physical and chemical properties and rhizosphere microbiomes (Buyer *et al*., 1999; Latour *et al*., 1999; Marschner *et al*., 2004), and plant yields generally perform better in soil types that are suitable for plant growth. Adding beneficial microbes to the soil can increase crop yields and suppress plant disease. Applying microbial products that contain *Bacillus subtilis* and *Trichoderma harzianum* in advance can boost potato yield and suppress potato common scab, which also increases the relative abundance of other beneficial bacteria such as *Burkholderiales* and *Pseudomonadales* in the rhizosphere (Wang *et al*., 2019).

However, given the complex plant‐related microbiome and low‐efficiency screening methods, finding and applying PGPR accurately to promote plant growth are difficult. Top‐down and synthetic community (SynCom) approaches provide high‐efficiency methods to find and apply PGPR. With the development of high‐throughput techniques, more microbiome structures of different plants are surveyed, including *Arabidopsis* (Lundberg *et al*., 2012; Schlaeppi *et al*., 2014), rice (Knief *et al*., 2012), bean (Perez‐Jaramillo *et al*., 2019), wheat (Donn *et al*., 2015), citrus (Xu *et al*., 2018), grape (Marasco *et al*., 2018) and maize (Kudjordjie *et al*., 2019). Top‐down approaches based on high‐throughput technology are used for the isolation of key members responsible for high‐yield and disease‐resistant phenotypes (Vorholt *et al*., 2017). For example, Proteobacteria, Firmicutes and Actinobacteria are detected as the key bacteria involved in the suppression of a fungal root pathogen in disease‐suppressive soils by coupling PhyloChip‐based metagenomics of the rhizosphere microbiome with culture‐dependent functional analyses (Mendes *et al*., 2011). *Flavobacteria* are enriched in rhizosphere microbiota of a variety of wilt‐resistant tomatoes. A Flavobacterium strain, namely, TRM1, is cultivated, which could suppress tomato wilt caused by *Ralstonia solanacearum* (Kwak *et al*., 2018). In addition, SynCom is created artificially by co‐culturing of select (two or more) species under (at least initially) well‐defined media (Großkopf and Soyer, 2014). Experimentally tractable SynCom approaches are used to verify the correlation of high‐throughput sequencing data and the interaction between plants and microbes (Vorholt *et al*., 2017; Liu *et al*., 2019). Whereas, artificial microbiome selection could obtain microbial superior properties that are beneficial to plants (Mueller and Sachs, 2015). Different bacterial SynCom have been organized in many studies to change plant phenotypes, such as high yield and disease resistance (Bai *et al*., 2015; Castrillo *et al*., 2017; Niu *et al*., 2017). SynCom can provide an effective and stable way for PGPR applications.

Garlic is a globally popular crop, which has a long history of planting, and plays an important role on people's dining tables (Rivlin, 2001). The garlic extract works against a broad range of plant pathogens, including bacteria and fungi (Curtis *et al*., 2004), and has the ability to inhibit the growth of oral pathogens (Bakri and Douglas, 2005). However, reports on the rhizosphere microbiome and PGPR in garlic rhizosphere are inadequate. Here, we hypothesized that the garlic rhizosphere microbial community is altered in different growth periods and different growth conditions. Moreover, the bacteria enriched in the rhizosphere in the bolting (April) and maturation (May) periods and good growth conditions are similar, and these bacteria are potential candidates of PGPR. In this study, the good growth conditions include fertile soil (loam, sandy loam) and agricultural practices with microbial products. We also hypothesized that these potential PGPR selected by top‐down approaches could be used to promote plant growth by SynCom approaches. To address these hypotheses, field experiments in Jinxiang county were conducted, where microbial products were applied to three soil types (sand, loam and sandy loam), and rhizosphere soil (RS) samples were collected during the three growth periods of garlic (resume growth, bolting and maturation) for amplicon sequencing. High‐throughput sequencing was used to reveal rhizosphere microbiome and the differential bacteria in the bolting and maturation periods and good growth conditions, which is an efficient top‐down approach for screening PGPR. SynCom approaches were used to verify the growth promotion effect of the selected PGPR.

## Results

### Garlic has high production in good growth conditions

**Fig. 1:**
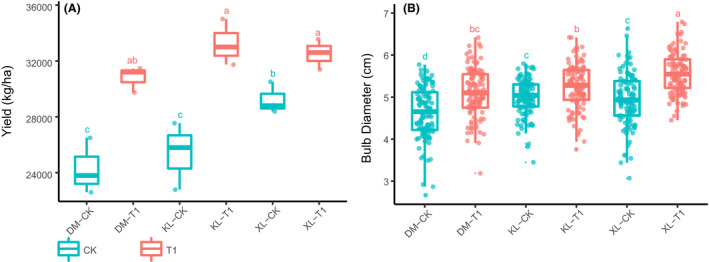
Garlic growth indicators of the harvest period in the field. The variation of yield (A *P* < 0.001, ANOVA, Tukey HSD) and bulb diameter (B *P* < 0.001, Kruskal–Wallis, Dunnetts) in different plots. Different letters indicate significantly different groups (LSD). XL, loam; KL, sandy loam; DM, sandy soil; CK, plots without microbial product; T1, plots with microbial product.

To explore the variation of garlic growth situation in different soil types and microbial product treatments, the yield and the diameter of bulbs reflecting the quality of garlic were measured during the harvest period of garlic. In addition, soil chemical characteristics are different in three soil types. The overall nutritional level of loam was higher than other soil types, and the nutritional level of sandy soil was the lowest (Table S1). Compared with sandy soil, loam and sandy loam belong to fertile soil. The results showed that garlic growth indicators (yield and bulb diameter) of the loam and sandy loam in plots without microbial product were significantly higher than those in sandy soil, and the indicators of the loam and sandy in plots with microbial product were also higher than those in the sandy soil. Compared with the plots without microbial product, the growth indicators (yield and bulb diameter) were significantly increased in the plots with microbial product in all each soil type (yield: *P* < 0.001, ANOVA, Tukey HSD; bulb diameter: *P* < 0.001, Kruskal–Wallis, Dunnetts) (Fig. 1, Data S1, Tables S2 and S3). In this study, the good growth conditions indicated fertile soil (loam, sandy loam) and agricultural practices with microbial products, and garlic under good growth conditions had higher growth indicators.

### Garlic rhizosphere microbiota have a distinct structure in different growth periods, soil types and treatments

A profile of bacterial and fungal communities was achieved to reveal the microbial community structure of garlic rhizosphere. Clean data of bacterial and fungal communities were acquired, and 1 866 039 merged sequence reads for the bacterial library and 1 419 773 merged sequence reads for the fungal library were obtained respectively. After strict filtering process, 1526 amplicon sequence variants (ASVs) were obtained from 83 samples for the bacterial library and 571 ASVs were obtained from 89 samples for the fungal library.

**Fig. 2:**
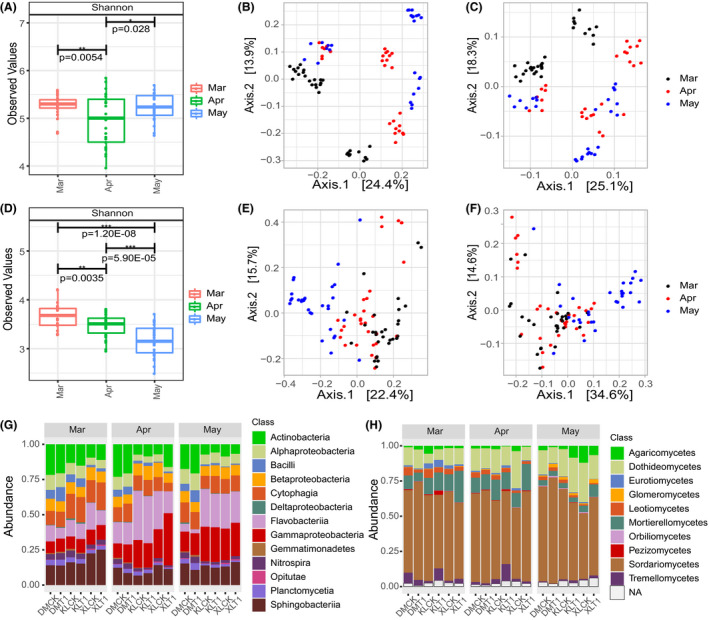
Alpha diversity, beta diversity and taxonomic profile of bacterial and fungal communities in three growth periods of garlic. A. ASV bacterial Shannon diversity index for three growth periods of garlic. B. PCoA of bacterial communities with Bray–Curtis distance in three growth periods. C. PCoA of bacterial communities with weighted UniFrac matrices in three growth periods. D. ASV fungal Shannon diversity index for three growth periods of garlic. E. PCoA of fungal communities with Bray–Curtis distance in three growth periods. F. PCoA of fungal communities with weighted UniFrac matrices in three growth periods. G. Relative abundance of bacterial classes of top 100 ASVs is shown in different groups. H. Relative abundance of fungal classes of top 100 ASVs is shown in different groups. Mar, resume growth period; Apr, bolting period; May, maturation period; XL, loam; KL, sandy loam; DM, sandy soil; CK, plots without microbial product; T1, plots with microbial product.

Garlic has distinct rhizosphere microbiota in different growth periods. By comparing alpha and beta diversity of microbiota in different growth periods, the bacterial Shannon diversity index was significantly lower in the bolting period compared with the resume growth and maturation periods (Fig. 2A, *P*‐value cut‐off = 0.05). Compared with the bolting and resume growth periods, the fungal ASV Shannon diversity index was significantly lower in the maturation period (Fig. 2D, *P*‐value cut‐off = 0.05). The principal coordinate analysis (PCoA) of Bray–Curtis distance and weighted UniFrac matrices showed that the distinct structure of bacterial (Table 1, Fig. 2B and C, permutational analysis of variance [PERMANOVA] by adonis, Bray–Curtis distance, *R* ^2^ = 0.19, *P* < 0.001, weighted UniFrac matrices, *R* ^2^ = 0.21, *P* < 0.001) and fungal (Table 1, Fig. 2E and F, PERMANOVA by adonis, Bray–Curtis distance, *R* ^2^ = 0.19, *P* < 0.001, Weighted UniFrac matrices, *R* ^2^ = 0.21, *P* < 0.001) rhizosphere microbiota in three growth periods formed separated clusters in the first two coordinate axes. All of the profiles indicated that rhizosphere microbiota changed with the growth of garlic. In addition, the relative abundance of bacterial and fungal classes of the top 100 most abundant ASVs in different garlic growth periods is shown in Fig. 2G and H.

**Table 1:** Bray–Curtis and weighted UniFrac matrices for bacterial and fungal microbiota were subjected to permutational analysis of variance (PERMANOVA) using the adonis test.

Dataset	Factor	Bray–Curtis distance		Weighted UniFrac	
		Bacteria (*R* ^2^)	Fungi (*R* ^2^)	Bacteria (*R* ^2^)	Fungi (*R* ^2^)
Whole	Growth Period	0.19***	0.19***	0.21***	0.21***
Mar	Soil Type	0.41***	0.29***	0.46***	0.26***
Mar‐DM	Treatment	0.23**	0.40**	0.24*	0.52**
Mar‐KL	Treatment	0.19**	0.29**	0.17**	0.24**
Mar‐XL	Treatment	0.30*	0.28*	0.36*	0.27*
Apr	Soil Type	0.43***	0.27***	0.47***	0.24***
Apr‐DM	Treatment	0.30*	0.23*	0.30*	0.2
Apr‐KL	Treatment	0.25**	0.14	0.23**	0.14
Apr‐XL	Treatment	0.45**	0.59**	0.48**	0.65*
May	Soil Type	0.50***	0.33***	0.52***	0.30***
May‐DM	Treatment	0.26*	0.31*	0.31*	0.26**
May‐KL	Treatment	0.29**	0.23*	0.33**	0.16
May‐XL	Treatment	0.34*	0.33*	0.34*	0.45**

Signif. codes: 0, ‘***’: 0.001, ‘**’: 0.01, ‘*’: 0.05, ‘.’ 0.1, ‘ ’: 1. *R* ^2^ for proportion of variation explained.

Microbiota dissimilarity assessment using 1000 permutations. Mar, resume growth period; Apr, bolting period; May, maturation period; XL, loam; KL, sandy loam; DM, sandy soil.

**Fig. 3:**
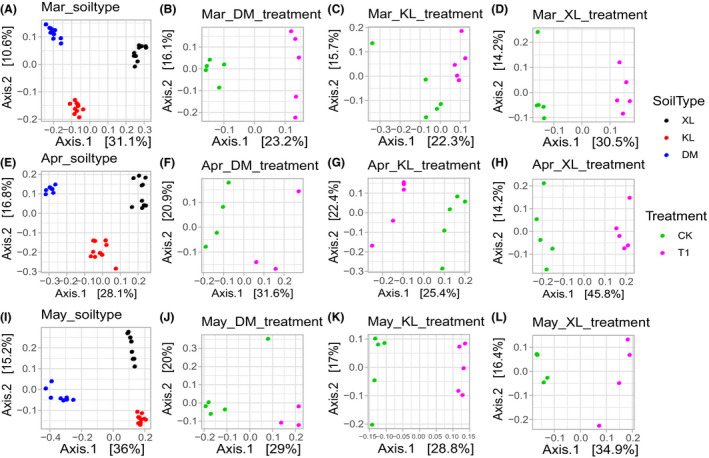
PCoA of bacterial microbiota using Bray–Curtis distance for soil type factor and treatment factor. (A, E, I) PCoA for soil type factor in different growth periods. (B–D) PCoA for treatment factor in different soil types in Mar. (F–H) PCoA for treatment factor in different soil types in Apr. (J–L) PCoA for treatment factor in different soil types in May. Mar, resume growth period; Apr, bolting period; May, maturation period; XL, loam; KL, sandy loam; DM, sandy soil; CK, plots without microbial product; T1, plots with microbial product.

**Fig. 4:**
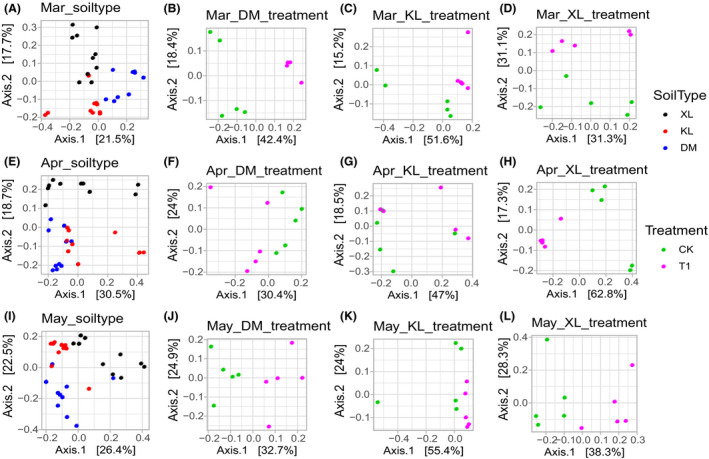
PCoA of fungal microbiota using Bray–Curtis distance for soil type factor and treatment factor. (A, E, I) PCoA for soil type factor in different growth periods. (B–D) PCoA for treatment factor in different soil types in Mar. (F–H) PCoA for treatment factor in different soil types in Apr. (J–L) PCoA for treatment factor in different soil types in May. Mar, resume growth period; Apr, bolting period; May, maturation period; XL, loam; KL, sandy loam; DM, sandy soil; CK, plots without microbial product; T1, plots with microbial product.

Garlic rhizosphere microbiota in different soil types and treatments were also distinct. The Mar, Apr and May datasets selected from the whole dataset were used to analyse the soil type factor. DM, KL and XL datasets from each growth period were used to analyse the treatment factor. The beta diversity and PCoA analyses using Bray–Curtis distance matrices showed that bacterial and fungal microbiota of different soil types in three growth periods datasets were separated in the first two coordinate axes. Treatment factors had good clustering in different soil type datasets and were separated in the first two coordinate axes (Table 1, Figs 3 and 4). For the Apr‐KL datasets (KL dataset selected from Apr dataset), no significant difference was found between plots with and without microbial products. To explore the bacterial and fungal microbiota structure of different factors in growth period and soil type datasets, we accepted PCoA analysis using weighted UniFrac matrices. The results were similar to those of Bray–Curtis distance matrices, but the treatment factors in Apr‐DM datasets did not show a significant difference (Table 1, Figs S1 and S2). A large variation was found in the rhizosphere microbiota of garlic with different soil types and treatments, and a strong correlation was observed between the structure of the microbiota in the whole dataset and soil chemistry (Fig. S3), which indicated that the soil type and treatment affected the rhizosphere microbiota of garlic.

### *Pseudomonas* was enriched in rhizosphere microbiota of the bolting and maturation periods and good growth conditions

Garlic rhizosphere microbiota in the bolting and maturation periods were distinguished from the resume growth period, and the rhizosphere microbiota in good growth conditions were also different from that in sandy soil and plots without microbial product. Paired comparison groups were used to detect differentially bacterial genus. Finally, eight comparison groups with more upregulated genus (> 7) of bacteria in the bolting and maturation periods and in good growth conditions were selected (Data S2). These eight comparison groups were the Apr_Mar, May_Mar (beta diversity of growth period factor in the whole datasets of bacteria, Table 1, PERMANOVA by adonis, Bray–Curtis distance, *R* ^2^ = 0.19, *P* < 0.001, weighted UniFrac matrices, *R* ^2^ = 0.21, *P* < 0.001), Mar.XL_DM (beta diversity of soil type factor in Mar datasets, Table 1, PERMANOVA by adonis, Bray–Curtis distance, *R* ^2^ = 0.41, *P* < 0.001, weighted UniFrac matrices, *R* ^2^ = 0.46, *P* < 0.001), Apr.XL_DM, Apr.KL_DM (beta diversity of soil type factor in Apr datasets, Table 1, PERMANOVA by adonis, Bray–Curtis distance, *R* ^2^ = 0.43, *P* < 0.001, weighted UniFrac matrices, *R* ^2^ = 0.47, *P* < 0.001), May.XL_DM, May.KL_DM (beta diversity of soil type factor in May datasets, Table 1, PERMANOVA by adonis, Bray–Curtis distance, *R* ^2^ = 0.50, *P* < 0.001, weighted UniFrac matrices, *R* ^2^ = 0.52, *P* < 0.001), and Apr.XL.T1_CK groups (beta diversity of treatment factor in Apr‐XL datasets, Table 1, PERMANOVA by adonis, Bray–Curtis distance, *R* ^2^ = 0.45, *P* < 0.001, weighted UniFrac matrices, *R* ^2^ = 0.48, *P* < 0.001), which had distinct beta diversity.

**Fig. 5:**
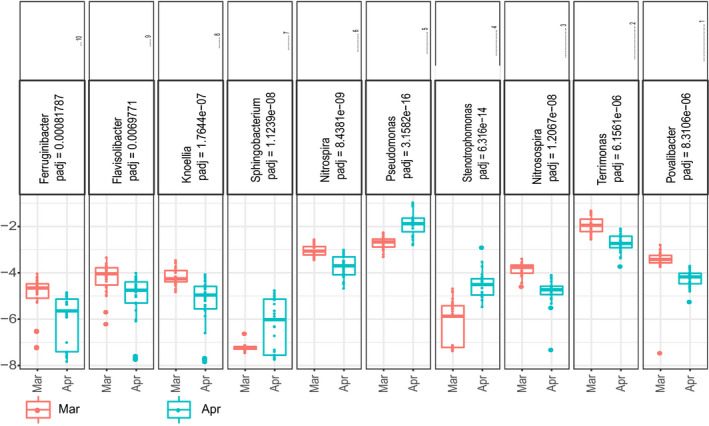
Top 10 differentially abundant genus of bacteria between Apr and Mar comparison groups in the whole datasets. Corresponding adjusted *P*‐values and rank of importance were detected by random forest classifier. Mar group is red; Apr group is blue. Mar, resume growth period; Apr, bolting period.

**Fig. 6:**
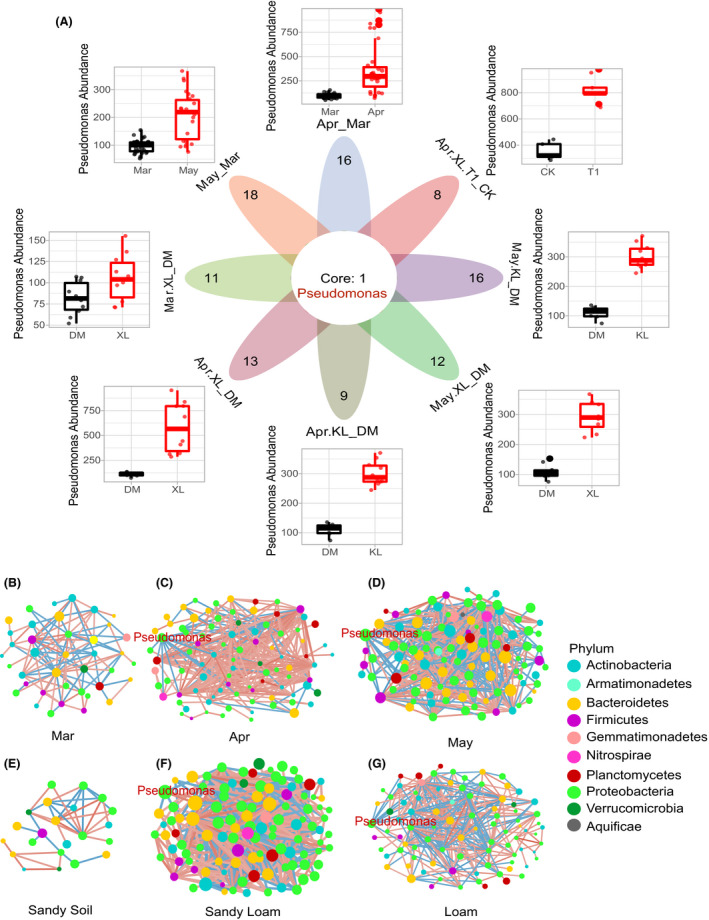
Bacterial genus enriched in eight comparison groups and co‐occurrence networks of Mar, Apr, May sandy soil, sandy loam and loam datasets. *Pseudomonas* was enriched in eight comparison groups under the late growth periods and good growth conditions, and the core plant growth‐promoting bacteria for garlic are *Pseudomonas* (A). Eight comparison groups have different colours, and the number of genus enriched in each comparison group is at the top of each oval. The eight accessory pictures around the Venn diagram represent the abundance difference of *Pseudomonas* in the eight comparison groups. Co‐occurrence networks of Mar (B), Apr (C), May (D), sandy soil (E), sandy loam (F) and loam (G) datasets. Compared with the network of Mar and sandy soil datasets, *Pseudomonas* plays more important role in the Apr, May, sandy loam and loam networks. Different colours of nodes represent different phyla of bacterial microbiota. Correlations between genus were expressed in different colour edges (positive correlation was represented as red edges; negative correlations were represented as blue edges), and the size of nodes indicated the abundance of genus. Nodes not assigned by genus and not connected to the main network were removed. Mar, resume growth period; Apr, bolting period; May, maturation period; XL, loam; KL, sandy loam; DM, sandy soil; CK, plots without microbial product; T1, plots with microbial product.

Compared with microbiota in the resume growth period, *Rhizobium*, *Pseudomonas*, *Sphingobacterium*, *Stenotrophomonas* and *Sphingopyxis* were enriched in the bolting and maturation periods (Data S2). Compared with microbiota in sandy soil in Mar, *Pseudomonas*, *Povalibacter*, *Flavobacterium*, *Pirellula*, *Flavisolibacter*, *Terrimonas* and *Stenotrophomonas* were enriched in loam plots in the resume growth period (Data S2). Compared with microbiota in sandy soil in the bolting period, *Massilia*, *Methylophilus*, *Pseudomonas* and *Flavobacterium* were enriched in sandy loam plots in the bolting period (Data S2), and *Methylotenera*, *Sphingorhabdus*, *Flavisolibacter*, *Stenotrophomonas*, *Pseudomonas*, *Flavobacterium*, *Terrimonas* and *Rhizobium* were enriched in loam plots in the bolting period (Data S2). Compared with microbiota in sandy soil in the maturation periods, *Chryseobacterium*, *Sphingopyxis*, *Sphingobacterium*, *Pedobacter*, *Cellvibrio*, *Sphingobium*, *Sphingorhabdus*, *Stenotrophomonas*, *Pseudomonas*, *Flavobacterium* and *Rhizobium* were enriched in sandy loam plots in the maturation period (Data S2). Compared with microbiota in sandy soil in the maturation period, *Cellvibrio*, *Sphingorhabdus*, *Stenotrophomonas*, *Pseudomonas*, *Flavobacterium* and *Rhizobium* were enriched in loam in the maturation period (Data S2). During the bolting period, compared with plots without a microbial product in loam, *Ensifer*, *Agromyces*, *Arthrobacter* and *Pseudomonas* were enriched in microbial product treatment plots in loam (Data S2). Bacterial genus enriched in eight comparison groups with more upregulated genus were compared. *Pseudomonas* was enriched in the bolting and maturation periods and in good growth conditions of the eight comparison groups (Fig. 6A). In addition, *Pseudomonas* was detected in the top 10 differentially abundant genus in most of the comparison groups (Fig. 5, Figs S4–S10) and was one of the important features among the differentially abundant genus (Fig. S11) in all comparison groups.

Compared with Mar and sandy soil datasets, co‐occurrence networks showed that Apr, May, sandy loam, and loam datasets had more top 0.01% genus, and microbiota of loam and Apr datasets had more complex interrelationships among different genera (Fig. 6B and g, Fig. S12, Data S3). The degree of *Pseudomonas* in Apr, May, sandy loam, and loam dataset networks was 18, 16, 5 and 20 respectively. The closeness centrality of *Pseudomonas* in Apr, May, sandy loam, and loam dataset networks was 0.054, 0.040, 0.064 and 0.067 respectively. The betweenness centrality of *Pseudomonas* in Apr, May, sandy loam and loam dataset networks was 240, 669, 9 and 248 respectively. By contrast, *Pseudomonas* was filtered by the co‐occurrence network parameters (r.threshold = 0.65, p.threshold = 0.01; nodes not assigned by genus and not connected to the main network were removed) in Mar and in sandy soil dataset networks (Fig. 6B and g, Fig. S12, Data S4).

### SynCom with six different *Pseudomonas* strains promote plant growth

**Fig. 7:**
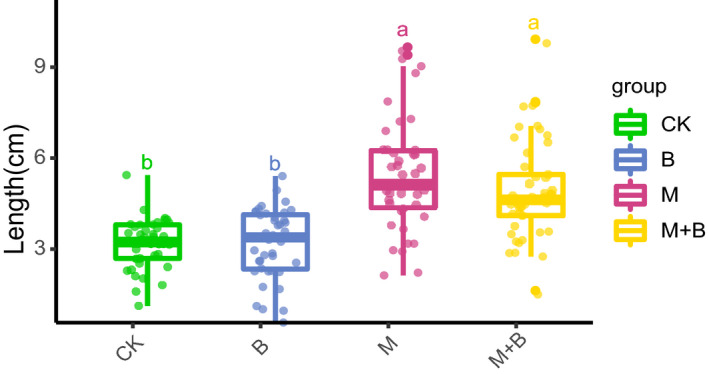
SynCom with six *Pseudomonas* sp. strains significantly promote plant growth. The variation of radish seedling length with three SynComs (*P* < 0.001, Kruskal–Wallis, Dunnetts). Different letters indicate significantly different groups (LSD). Group CK used sterile water for the negative control. Group M consisted of six *Pseudomonas* sp. strains with different phylogenetic names mixed in equal proportions. Group M + B added B8‐7 strain to the M group in equal proportions. B group only contains B8‐7 strain that was used to be the positive control.

Three SynComs were established, and the good growth‐promoting effect of SynCom with different *Pseudomonas* sp. isolated from RS was verified on radish seedlings. The length of radish seedlings in the M and M + B groups was significantly higher than that in the CK and B groups (Fig. 7, Data S5, Fig. S13, Table S5, *P* < 0.001, Kruskal–Wallis, Dunnetts). The B8‐7 strain did not promote the growth of radish seedlings and did not significantly affect the growth‐promoting effect of the M group.

## Discussion

Plants not only actively influence the microbiome structure in different root niches by a multistep model (Edwards *et al*., 2015) but also increase the abundance of beneficial communities in the rhizosphere at specific growth periods (Zhang *et al*., 2018). Garlic in Jinxiang County grows slowly in March (resume growth period) and is harvested in May (maturation period). The bolting period plays a key role in the growth of garlic. During this period, the leaf area reaches its maximum, and the bulbs swell with the growth of garlic in Jinxiang. Fertile soil (loam, sandy loam) is also important to garlic growth, and microbial products could promote garlic growth in all three soil types (Fig. 1, Data S1). Moreover, Microbial inheritance from the seed to root and from the first generation of plants to the next, and a term called ‘microbiota‐induced soil inheritance (MISI)’ explains that the recruitment and accumulation of microbiota by biotic and abiotic stresses affect plant immunity in the next generation through plant–soil feedback and soil memory (Kong *et al*., 2019).

The goal of this study was to determine (i) whether the garlic rhizosphere microbial community changed in different growth periods and different growth conditions, and (ii) whether the bacteria enriched in the bolting (April) period, maturation (May) period and good growth conditions are PGPR, and (iii) whether these enriched bacteria promote plant growth by SynCom approaches. Through high‐throughput sequencing, rhizosphere microbiota of garlic in different growth periods, soil types and treatments are revealed. The main bacteria in garlic rhizosphere at the class level were Actinobacteria, Alphaproteobacteria, Bacilli, Betaproteobacteria, Cytophagia, Flavobacteriia, Gammaproteobacteria and Sphingobacteriia, and the main fungi at the class level were Dothideomycetes, Mortierellomycetes, Sordariomycetes and Tremellomycetes (Fig. 2G and H).

Microbiota changed during the growth periods of garlic, which is a dynamic process. *Sphingopyxis*, *Sphingobium*, *Sphingobacterium*, *Rhizobium*, *Pseudomonas*, *Lacibacter* and *Cellvibrio* were recruited in the rhizosphere in the bolting and maturation periods (Data S2). In addition, the rhizosphere microbiota in loam and sandy loam are different from that in sandy soil, and *Pseudomonas*, *Flavobacterium* and *Hydrophile* are enriched in loam and sandy loam soil (Data S2). Some of the enriched bacteria are beneficial for plant growth and the environment. For example, *Rhizobium* and *Flavobacterium* have the characteristics that promote plant growth (Antoun *et al*., 1998; Soltani *et al*., 2010). *Sphingopyxis*, *Sphingobium* and *Sphingobacterium* have the ability to degrade a broad range of mono‐ and polycyclic aromatic compounds (Kertesz and Kawasaki, 2010). Moreover, microbial products are becoming popular as substitutes for chemical pesticides. Studies have shown that adding microbial products containing a large amount of beneficial bacteria into a cultivated field could promote plant growth and inhibit disease occurrence, and some of the microbial products increase the number of *Pseudomonas* (Shen, 1997; Wang *et al*., 2019).

In the rhizosphere, the bacteria enriched in the bolting and maturation periods and in good growth conditions are similar, and *Pseudomonas* is significantly enriched. In the eight comparison groups, the number of ASVs belong to *Pseudomonas* is higher under the late growth periods and high‐yield conditions (Fig. 6A). Due to the strict the co‐occurrence network parameters, *Pseudomonas* did not appear in Mar and in sandy soil dataset networks. However, network features of *Pseudomonas* ranked high in the co‐occurrence network of Apr, Mar, sandy loam and loam datasets, which indicated that *Pseudomonas* was important in rhizosphere microbiota in the bolting and maturation periods and in fertile soil types (Fig. 6B and G, Data S4). The co‐occurrence network analysis shows that, with the growth of garlic, the interaction of bacterial communities in the rhizosphere became more and more complex, and the interaction between *Pseudomonas* and other bacteria in the rhizosphere bacterial network increased. Compared with the network of sandy soil dataset, the changes of *Pseudomonas* in fertile soil also showed the same trend as the growth periods. In addition, most of the interactions between *Pseudomonas* and other bacteria are negative in Apr, May, sandy loam and loam dataset networks (Fig. 6B and G, Data S3), which indicates that *Pseudomonas* might gain survival advantages by seizing the niche of other bacteria to promote garlic growth and suppress pathogenic microbes. *Pseudomonas* made available inorganic nutrients to the plants favouring plant growth and then the benefit of the interaction is common (Garciasalamanca *et al*., 2013). Previous studies have shown that *Pseudomonas* can promote plant growth and inhibit the occurrence of many plant diseases (Preston, 2004; Sandhya *et al*., 2010; Hu *et al*., 2016). *Pseudomonas piscum* can inhibit growth and virulence of the plant pathogenic fungus *Fusarium graminearum* (Chen *et al*., 2018).

The results of different bacterial and co‐occurrence network analyses indicate that *Pseudomonas* might play an important role in garlic growth, and *Pseudomonas* is a potential PGPR of garlic. Furthermore, increasing *Pseudomonas* community richness is beneficial to the biomass and nutrient content of plant, and compared with single‐strain inoculants, multi‐strain microbial inoculants can promote plant growth more reliably and effectively (Hu *et al*., 2017). Thus, a mixed SynCom with six *Pseudomonas* strains isolated from the rhizosphere of garlic were constructed. The experiment of radish seedlings in the incubator proved that the SynCom composed of six *Pseudomonas* strains has an evident plant growth‐promoting effect (Fig. 7).

In conclusion, our results support the hypothesis that the garlic rhizosphere microbial community changed in different growth periods and different growth conditions, and the bacteria enriched in the bolting (April) period, maturation (May) period and good growth conditions are PGPR which can promote plant growth by SynCom approaches. We demonstrated that *Pseudomonas* was enriched in rhizosphere microbiota of the bolting and maturation periods and good growth conditions which was a key PGPR in the rhizosphere of garlic, and SynCom with six *Pseudomonas* strains isolated from the garlic rhizosphere can be constructed to promote plant growth. The rhizosphere microbial community is relatively stable, thus the growth promotion effect of the exogenous bacterial community is affected by the native rhizosphere microbial community of crops in farmland, which is also affected by the climate, soil chemistry and agricultural practices. However, *Pseudomonas* exists in the rhizosphere of most plants. For example, *Pseudomonas* belongs to the core community of maize (Walters *et al*., 2018). The SynCom, which has six *Pseudomonas* strains isolated from the rhizosphere of garlic, can promote the growth of radish seedlings. Thus the SynCom in this study might have great potential to survive in RS of multiple crops and play a role in promoting plant growth, which has broad application prospects.

In this study, a top‐down approach was used to identify *Pseudomonas* as PGPR quickly and accurately, which includes eight comparison groups that have high selection pressures because of large differences in garlic growth conditions and co‐occurrence network analysis. This top‐down approach could be combined with Known Media Database (Oberhardt *et al*., 2015) that can predict media by an organism 16S rDNA sequence to facilitate cultivation efforts, avoid large‐scale microbes isolation and cultivation and improve the efficiency of obtaining PGPR. Furthermore, SynCom approaches not only provide insights into how plants affect their microbial community and how the microbiome affects plant growth and health but also provide high operability and application value for agricultural production to promote plant growth, resist plant diseases, reduce the use of chemical pesticides and improve soil quality. The top‐down approaches based on high‐throughput sequencing, media prediction technology and SynCom approaches can be combined to serve as the foothold for precision agriculture and green agriculture, improve the formula of microbial products accurately and promote personalization of microbial products.

## Experimental procedures

### Experimental design and sample collection

This study was conducted in loam (XL), sandy loam (KL) and sandy soil (DM) in Jinxiang County, Shandong Province, China, in 2017. For each soil type, a microbial product (Sino Green Agri‐Biotech, Beijing, China) was used in garlic, which was designed as a treatment plot (T1). The size of each plot with and without microbial product is 6 m × 100 m. The microbial product primarily contained *Bacillus subtilis* strain znjdf1 (strain accession number: CGMCC NO.7850), *Trichoderma harzianum* strain znlkhc1 (strain accession number: CGMCC NO.7861) and an inert carrier (diatomaceous earth). Seed dressing was adopted, and the product concentration was 75 kg ha^−1^ in each treatment plot. Except for the soil type and treatment of the microbial product, similar locally agricultural practices and variety of garlic, namely, Jinxiang purple peel garlic, were used in all plots. In the resume growth (March), bolting (April) and maturation periods (May), the non‐RS that was not tightly attached to the garlic roots was removed by vigorously shaking, and then, the soil attached to the root surface (approximately 1–2 mm) was carefully separated and collected by brush as RS. Five replicate samples were collected in each plot, and each replicate consisted of the pooled RS obtained from 15 plants. The RS was placed into sterile sampling bags and then placed on ice and transported to the laboratory within 12 h. To remove garlic roots and other impurities, the RS was filtered with a 200‐mesh sieve. After thorough mixing, the RS was stored at −80°C until DNA was extracted.

### Soil chemical analysis

Bulk soil of each plot in the resume growth, bolting and maturation growth periods was filtered by a sieve, and then, the filtered soil was subjected to soil chemical analysis, including pH, soil organic matter, total nitrogen (Ntotal), available phosphorus (Pavailable), available potassium (Kavailable), available manganese (Mnavailable), available iron (Feavailable) and available copper (Cuavailable) (Table S1).

### Plant growth conditions in the field

During the harvest of garlic in May, the yield of garlic was measured in three scattered and random regions. One hundred bulbs were randomly selected in each plot, and the bulb diameter was measured with a vernier caliper (Data S1).

### DNA extraction, polymerase chain reaction (PCR) amplification and sequencing

The total DNA for each soil sample was extracted using the FastDNA SPIN Kit for soil (MP Biomedicals) according to the instruction manual. The quality and concentration of DNA were measured by agarose gel electrophoresis and a spectrophotometer (NanoDrop ND‐2000, Wilmington, DE, USA) and were subsequently diluted to 5 ng·µl^−1^. Bacterial and fungal sequencing libraries from 90 DNA samples were prepared. The V3–V4 region of bacterial 16S rRNA gene was amplified by PCR using primers 515F (5′‐GTGCCAGCMGCCGCGGTAA‐3′) and 909R (5′‐CCCCGYCAATTCMTTTRAGT‐3′). The forward primer (515F) had a sample‐specific 12‐bp barcode that was used to distinguish samples. For the amplification of the fungal ITS1 region, ITS1 (5′‐CTTGGTCATTTAGAGGAAGTAA‐3′) and ITS2 (5′‐TGCGTTCTTCATCGATGC‐3′) primers with barcode were used. In addition, negative controls (no template was added) were used to detect the presence of contaminating sequences in the reagents and process of operation and confirmed by gel electrophoresis (1.5% agarose gel, 120 V, 30 min). If no amplification was visible in the negative control, then the PCR products were separated on a 2% agarose gel to extract the band of the expected size using the QIAquick Gel Extraction Kit (QIAGEN, Germany). The final concentration of the amplicon libraries was determined by Qubit (Thermo Fisher Scientific, USA), and amplicon libraries were mixed at equal moles for deep sequencing. Afterwards, the amplicon libraries of bacteria were applied to the Novaseq 6000 platform (Illumina) for sequencing, and the amplicon libraries of fungi were subjected to sequencing on the Miseq PE250 system (Illumina).

### Sequence data and statistical analysis

After removing barcodes, the dada2 package (Callahan *et al*., 2016) (v.1.12.1) was used to preprocess and construct the ASV table. Taxonomic assignments for the clustered ASVs were performed using the RDP trainset 16/release 11.5 for bacteria (Cole *et al*., 2014) and the UNITE database version 8.0 for fungal ASVs (Abarenkov *et al*., 2010). The phyloseq package (McMurdie and Holmes, 2013) (v.1.28.0) was used for downstream analysis of the ASV table. Furthermore, low‐abundance samples (samples with less than 3000 reads) were excluded. Rarefying was performed using phyloseq. The statistical analyses were implemented in R (R Core Team, 2013) (v.3.6.2). The whole dataset was divided into the Mar, Apr and May datasets (growth period datasets), and the sandy soil, sandy loam and loam (soil type datasets) datasets were selected from each growth period dataset (Table 1). Alpha diversity analysis was carried out using the microbiomeSeq package (Ssekagiri *et al*., 2018) (v.0.1). For beta diversity and partitioning of variance, Bray–Curtis and weighted UniFrac matrices for bacterial and fungal microbiota were subjected to PERMANOVA using the adonis test in the vegan package (Oksanen *et al*., 2007) (v.2.5.5), and unconstrained PCoA was used for visualization. CAP analysis based on Bray–Curtis distance was used to explore the contribution of environmental factors of soil chemistry to differences in bacterial and fungal microbiota. Constrained ordination was used to detect how environmental variables of soil chemistry are associated with changes in microbiota, and the ordination axes were constrained to linear combinations of environmental variables. Then, the environmental scores were plotted on the ordination. Differential abundance analysis was performed using the DESeq2 package (Love *et al*., 2014) (v.1.24.0). To find the most important feature among the differentially expressed bacterial genus, the microbiomeSeq package was used to detect the top 10 differentially abundant genus of bacteria among different comparison groups, and mean decrease accuracy values of differentially abundant genus were calculated. In addition, the sandy soil, sandy loam and loam datasets were selected from the whole dataset of bacteria for co‐occurrence network analysis. Co‐occurrence networks of top 0.01% abundant genus were constructed (r.threshold = 0.65, p.threshold = 0.01), and network properties were calculated using the igraph packages (Csardi and Nepusz, 2006) (v.1.2.4.1). Cytoscape was used to optimize and adjust the networks.

### SynCom and the verification of the plant growth‐promoting effect

Rhizosphere soil samples were selected on the basis of soil characters, and then, 263 garlic rhizosphere isolates were isolated from those samples. The bacterial DNA from each individual isolate was extracted, and then, the 16S rRNA gene was amplified by the primers 63F (5′‐CAGGCCTAACACATGCAAGTC‐3′) and 1387R (5′‐GGGCGGWGTGTACAAGGC‐3′). BLAST in the NCBI database (Sherry *et al*., 2001) was used to obtain the phylogenetic names of these isolates. Each bacterial suspension cultured by shaking in Luria–Bertani medium (LB) was mixed with sterilized 30% glycerol at the ratio of 1:1 and stored at −20°C for long‐term preservation. After analysis based on Illumina‐sequenced database and sequence alignment of isolated bacteria, six *Pseudomonas* sp. strains with different phylogenetic names and B8‐7 strain were selected to form different SynCom groups (Tables S5 and S6). The M group contained six *Pseudomonas* sp. strains with different phylogenetic names mixed in equal proportions, which probably belonged to *Pseudomonas cedrina*, *Pseudomonas baetica*, *Pseudomonas migulae*, *Pseudomonas fluorescens*, *Pseudomonas reinekei* and *Pseudomonas frederiksbergensis* (Tables S5 and S6, Data S6). In addition, the B group only contained B8‐7 strain, which probably belonged to *Bacillus simplex* (Tables S5 and S6, Data S6) and was used as the positive control, and sterile water was used for the negative control. B8‐7 strain was added to the M group in equal proportions as the M + B group. Bacterial strains were cultured (28°C for six *Pseudomonas* strains, 37°C for B8‐7, 18 mm × 180 mm glass tubes, shaking at 200 r.p.m.) in 10 ml LB for 2 days. After centrifugation, each strain was washed three times with sterile water quickly. The OD_600_ of each strain was diluted to 0.1–0.2 with sterile water, and then, 5 ml suspensions of each kind of strains were mixed into certain groups of SynCom according to the experimental design. To detect the plant growth‐promoting effect of SynComs quickly, radish seedlings were used. Radish seeds were surface sterilized in 3% NaCIO solution three times for 1 min, washed three times with sterile water and placed in sterile 100 mm × 100 mm petri dish, which contained a sterile filter paper filled with sterile water (26°C, 12 h), for germination. Seeds with similar growth conditions were selected and immersed in suspensions of different SynCom groups (26°C, 2 h). Each petri dish contained five seeds, and each SynCom group had three technical repeats and three biological repeats. Plants were grown at 26°C and 16 h light in an incubator. After 2 days, seedling length was measured to evaluate growth condition.

## Conflict of interest

None of the authors have any conflict of interest.

## Data and code availability

Illumina amplicon data have been submitted to SRA under PRJNA631473. The scripts used in the microbiome analysis are available in GitHub under https://github.com/rnnnnna/GRMP.

## Supporting information


**Fig. S1.** PCoA of bacterial microbiota using weighted UniFrac matrice for soil type factor and treatment factor. a,e,i PCoA for soil type factor in different growth periods. b–d PCoA for treatment factor in different soil types in Mar. f–h PCoA for treatment factor in different soil types in Apr. j–l PCoA for treatment factor in different soil types in May. Mar, resume growth period; Apr, bolting period; May, maturation period; XL, loam; KL, sandy loam; DM, sandy soil; CK, plots without microbial product; T1, plots with microbial product.
**Fig. S2.** PCoA of fungal microbiota using weighted UniFrac matrice for soil type factor and treatment factor. a,e,i PCoA for soil type factor in different growth periods. b–d PCoA for treatment factor in different soil types in Mar. f–h PCoA for treatment factor in different soil types in Apr. j–l PCoA for treatment factor in different soil types in May. Mar, resume growth period; Apr, bolting period; May, maturation period; XL, loam; KL, sandy loam; DM, sandy soil; CK, plots without microbial product; T1, plots with microbial product.
**Fig. S3.** CAP analysis based on Bray–Curtis distance for bacterial and fungal microbiota in whole dataset. a The contribution of environmental factors of soil chemistry to differences in bacteria microbiota. b The contribution of environmental factors of soil chemistry to differences in fungal microbiota. XL, loam; KL, sandy loam; DM, sandy soil; CK, plots without microbial product; T1, plots with microbial product.
**Fig. S4.** Top10 differentially abundant genus of bacteria between Mar and May comparison groups in the whole datasets. Corresponding adjusted *P*‐values and rank of importance were detected by random forest classifier. Mar groups is red, May groups is blue. Mar, resume growth period; May, maturation period.
**Fig. S5.** Top10 differentially abundant genus of bacteria between XL and DM comparison groups in the Mar datasets. Corresponding adjusted *P*‐values and rank of importance were detected by random forest classifier. XL groups is red, DM groups is blue. Mar, resume growth period; XL, loam; DM, sandy soil.
**Fig. S6.** Top10 differentially abundant genus of bacteria between KL and DM comparison groups in the Apr datasets. Corresponding adjusted *P*‐values and rank of importance were detected by random forest classifier. KL groups is red, DM groups is blue. Apr, bolting period; KL, sandy loam; DM, sandy soil.
**Fig. S7.** Top10 differentially abundant genus of bacteria between XL and DM comparison groups in the Apr datasets. Corresponding adjusted *P*‐values and rank of importance were detected by random forest classifier. XL groups is red, DM groups is blue. Apr, bolting period; XL, loam; DM, sandy soil.
**Fig. S8.** Top10 differentially abundant genus of bacteria between KL and DM compared groups in the May datasets. Corresponding adjusted *P*‐values and rank of importance were detected by random forest classifier. KL groups is red, DM groups is blue. May, maturation period; KL, sandy loam; DM, sandy soil.
**Fig. S9.** Top10 differentially abundant genus of bacteria between XL and DM comparison groups in the May datasets. Corresponding adjusted *P*‐values and rank of importance were detected by random forest classifier. XL groups is red, DM groups is blue. May, maturation period; XL, loam; DM, sandy soil.
**Fig. S10.** Top10 differentially abundant genus of bacteria between T1 and CK comparison groups in the Apr–XL datasets. Corresponding adjusted *P*‐values and rank of importance were detected by random forest classifier. CK groups is red, T1 groups is blue. Apr, bolting period; XL, loam; CK, plots without microbial product; T1, plots with microbial product.
**Fig. S11.** Mean decrease accuracy values of differentially abundant genus which can provide a stand visual representation of important features obtained by random forest classifer in different comparison groups. a Apr and Mar comparison groups in the whole datasets. b May and Mar comparison groups in the whole datasets. c XL and DM comparison groups in the Mar datasets. d KL and DM comparison groups in the Apr datasets. e KL and DM comparison groups in the Apr datasets. f KL and DM comparison groups in the May datasets. g XL and DM comparison groups in the May datasets. h T1 and CK comparison groups in the Apr‐XL datasets. Mar, resume growth period; Apr, bolting period; May, maturation period; XL, loam; KL, sandy loam; DM, sandy soil; CK, plots without microbial product; T1, plots with microbial product.
**Fig. S12.** Co‐occurrence networks of Mar, Apr, May sandy soil, sandy loam and loam datasets. Co‐occurrence networks of Mar (a), Apr (b), May(c) sandy soil (d), sandy loam(e) and loam (f) datasets. Different colours of nodes represent different phylum of bacteria microbiota. Correlations between genus were expressed in different colors edges (positive correlation were represented as red edges, negative correlations were represented as blue edges), and the size of nodes indicated the abundance of genus. Mar, resume growth period; Apr, bolting period; May, maturation period.
**Fig. S13.** SynCom promote radish seedlings growth. Group CK used sterile water for the negative control. Group M consisted of six *Pseudomonas* sp. strains with different phylogenetic names mixed in equal proportions.Group M+B added B8‐7 strain to M group in equal proportions. B group only contains B8‐7 strain that was used to be the positive control.
**Table S1.** Soil chemical situation in different growth periods, soil types and treatments. Mar, resume growth period; Apr, bolting period; May, maturation period; XL, loam; KL, sandy loam; DM, sandy soil; CK, plots without microbial product; T1, plots with microbial product.
**Table S2.** Tukey_HSD table of garlic yield. XL, loam; KL, sandy loam; DM, sandy soil.
**Table S3.** Dunnetts test table of garlic bulb diameter. Dunn's test of multiple comparisons using rank sums : holm. XL, loam; KL, sandy loam; DM, sandy soil; CK, plots without microbial product; T1, plots with microbial product.
**Table S4.** Dunnetts test table of radish seedlings length. Dunn's test of multiple comparisons using rank sums : holm. Group CK used sterile water for the negative control. Group M consisted of six Pseudomonas sp. strains with different phylogenetic names mixed in equal proportions.Group M+B added B8‐7 strain to M group in equal proportions. B group only contains B8‐7 strain that was used to be the positive control.
**Table S5.** Taxonomy of bacterial isolates that make up the synthetic community. Phylogenetic names of all 263 isolates which isolated from garlic rhizosphere soil was obtained by blast against NCBI comparison. Six Pseudomonas strains with different phylogenetic names and a Bacillus strain were selected to compose different synthetic community groups.
**Table S6.** 16S rRNA gene sequence of strains in SynComs

**Data S1.** Garlic bulb diameter and yield in May. May, maturation period; XL, loam; KL, sandy loam; DM, sandy soil; CK, plots without microbial product; T1, plots with microbial product.

**Data S2.** Differentially bacterial genus in eight comparison groups. Mar, resume growth period; Apr, bolting period; May, maturation period; XL, loam; KL, sandy loam; DM, sandy soil; CK, plots without microbial product; T1, plots with microbial product.

**Data S3.** Outputs of co‐occurrence networks in igraph object . Mar, resume growth period; Apr, bolting period; May, maturation period; XL, loam; KL, sandy loam; DM, sandy soil.

**Data S4.** Node properties of co‐occurrence networks.Mar, resume growth period; Apr, bolting period; May, maturation period; XL, loam; KL, sandy loam; DM, sandy soil.

**Data S5.** The radish seedlings length(cm) with different SynComs groups. Group CK used sterile water for the negative control. Group M consisted of six Pseudomonas sp. strains with different phylogenetic names mixed in equal proportions.Group M+B added B8‐7 strain to M group in equal proportions. B group only contains B8‐7 strain that was used to be the positive control.

**Data S6.** Taxonomy of bacterial isolates that make up the synthetic community. Taxonomy of all 263 isolates that isolated from garlic rhizosphere soil was obtained by NCBI comparison and phylogenetic analysis in MEGA5. Six Pseudomonas strains with different species and a Bacillus simplex strain were selected to compose different synthetic community groups.
