# Supplementary material for: Synthetic community with six Pseudomonas strains screened from garlic rhizosphere microbiome promotes plant growth
Source: Microb Biotechnol. 2020 Aug 6;14(2):488–502. doi: 10.1111/1751-7915.13640 (PMC7936309; doi:10.1111/1751-7915.13640)
Supplement: Supplementary file 1 — Fig. S1. PCoA of bacterial microbiota using weighted UniFrac matrice for soil type factor and treatment factor. a,e,i PCoA for soil type factor in different growth periods. b–d PCoA for treatment factor in different soil types in Mar. f–h PCoA for treatment factor in different soil types in Apr. j–l PCoA for treatment factor in different soil types in May. Mar, resume growth period; Apr, bolting period; May, maturation period; XL, loam; KL, sandy loam; DM, sandy soil; CK, plots without microbial product; T1, plots with microbial product. Fig. S2. PCoA of fungal microbiota using weighted UniFrac matrice for soil type factor and treatment factor. a,e,i PCoA for soil type factor in different growth periods. b–d PCoA for treatment factor in different soil types in Mar. f–h PCoA for treatment factor in different soil types in Apr. j–l PCoA for treatment factor in different soil types in May. Mar, resume growth period; Apr, bolting period; May, maturation period; XL, loam; KL, sandy loam; DM, sandy soil; CK, plots without microbial product; T1, plots with microbial product. Fig. S3. CAP analysis based on Bray–Curtis distance for bacterial and fungal microbiota in whole dataset. a The contribution of environmental factors of soil chemistry to differences in bacteria microbiota. b The contribution of environmental factors of soil chemistry to differences in fungal microbiota. XL, loam; KL, sandy loam; DM, sandy soil; CK, plots without microbial product; T1, plots with microbial product. Fig. S4. Top10 differentially abundant genus of bacteria between Mar and May comparison groups in the whole datasets. Corresponding adjusted P‐values and rank of importance were detected by random forest classifier. Mar groups is red, May groups is blue. Mar, resume growth period; May, maturation period. Fig. S5. Top10 differentially abundant genus of bacteria between XL and DM comparison groups in the Mar datasets. Corresponding adjusted P‐values and rank of importance were detected by rand [file MBT2-14-488-s001.docx]

**Synthetic Community with Six *Pseudomonas* Strains Screened from Garlic Rhizosphere Microbiome Promotes Plant Growth**

Supplementary Material.

${Lubo Zhuang}^{\text{a}}$, ${Yan Li}^{\text{a}}$, ${Zhenshuo Wang}^{\text{a}}$, ${Yue Yu}^{\text{a}}$, ${Nan Zhang}^{\text{a}}$, ${Chang Yang}^{\text{a}}$, ${Qingchao Zeng}^{\text{a}}$, $\text{Qi Wang}^{\text{a*}}$

**This file includes:**

Supplementary Figures S1–S13:

Fig. S1, PCoA of bacterial microbiota using weighted UniFrac matrice for soil type factor and treatment factor.

Fig. S2, PCoA of fungal microbiota using weighted UniFrac matrice for soil type factor and treatment factor.

Fig. S3, CAP analysis based on Bray–Curtis distance for bacterial and fungal microbiota in whole dataset.

Fig. S4, Top10 differentially abundant genus of bacteria between May and Mar comparison groups in the whole datasets.

Fig. S5, Top10 differentially abundant genus of bacteria between XL and DM comparison groups in the Mar datasets.

Fig. S6, Top10 differentially abundant genus of bacteria between KL and DM comparison groups in the Apr datasets.

Fig. S7, Top10 differentially abundant genus of bacteria between XL and DM comparison groups in the Apr datasets.

Fig. S8, Top10 differentially abundant genus of bacteria between KL and DM compared groups in the May datasets.

Fig. S9, Top10 differentially abundant genus of bacteria between XL and DM compared groups in the May datasets.

Fig. S10, Top10 differentially abundant genus of bacteria between T1 and CK compared groups in the Apr-XL datasets.

Fig. S11, Mean decrease accuracy values of differentially abundant genus which can provide a stand visual representation of important features obtained by random forest classifer in different comparison groups.

Fig. S12, Co-occurrence networks of Mar, Apr, May sandy soil, sandy loam and loam datasets.

Fig. S13, SynCom promote radish seedlings growth.

Supplementary Tables S1**–**S6:

Table S1, Soil chemical situation in different growth periods, soil types and treatments.

Table S2, Tukey_HSD table of garlic yield.

Table S3, Dunnetts test table of garlic bulb diameter.

Table S4, Dunnetts test table of radish seedlings length

Table S5, Taxonomy of bacterial isolates that make up the synthetic community.

Table S6, 16S rRNA gene sequence of strains in SynComs

**Supplementary Figures**

**
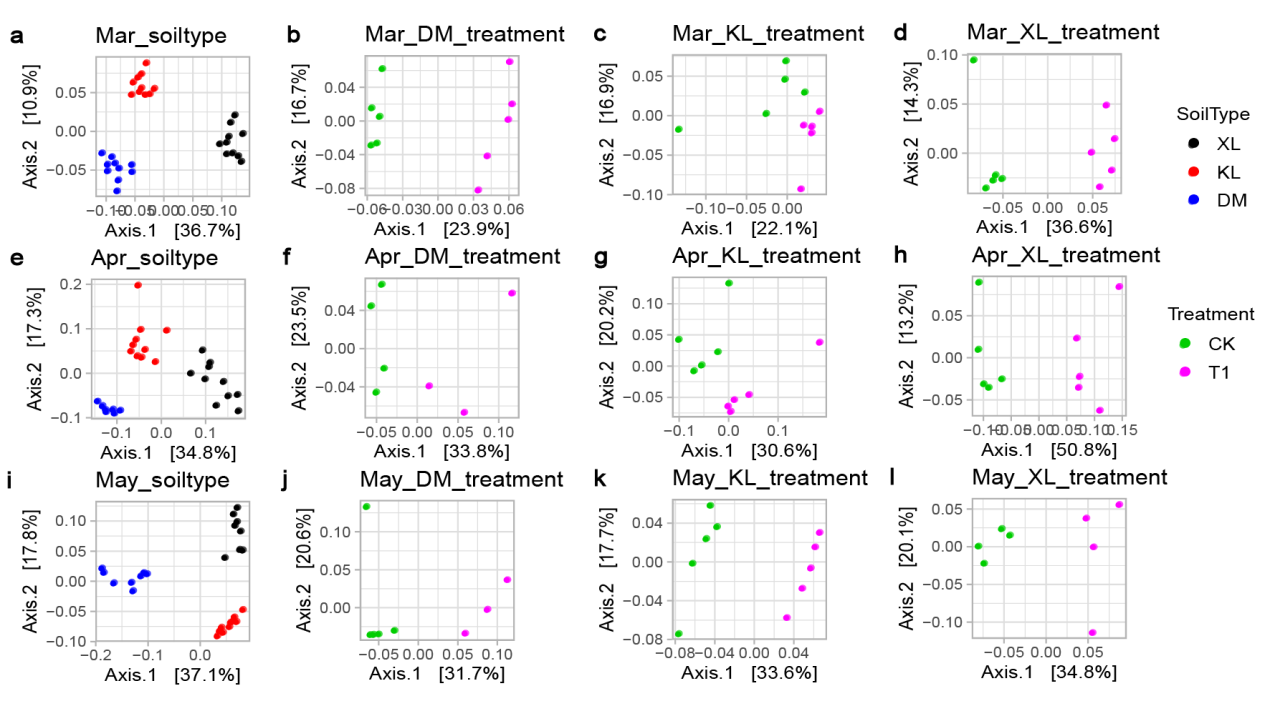
**

**Supplementary Fig. S1 PCoA of bacterial microbiota using weighted UniFrac matrice for soil type factor and treatment factor. a,e,i** PCoA for soil type factor in different growth periods. **b–d** PCoA for treatment factor in different soil types in Mar. **f–h** PCoA for treatment factor in different soil types in Apr. **j–l** PCoA for treatment factor in different soil types in May. Mar, resume growth period; Apr, bolting period; May, maturation period; XL, loam; KL, sandy loam; DM, sandy soil; CK, plots without microbial product; T1, plots with microbial product.


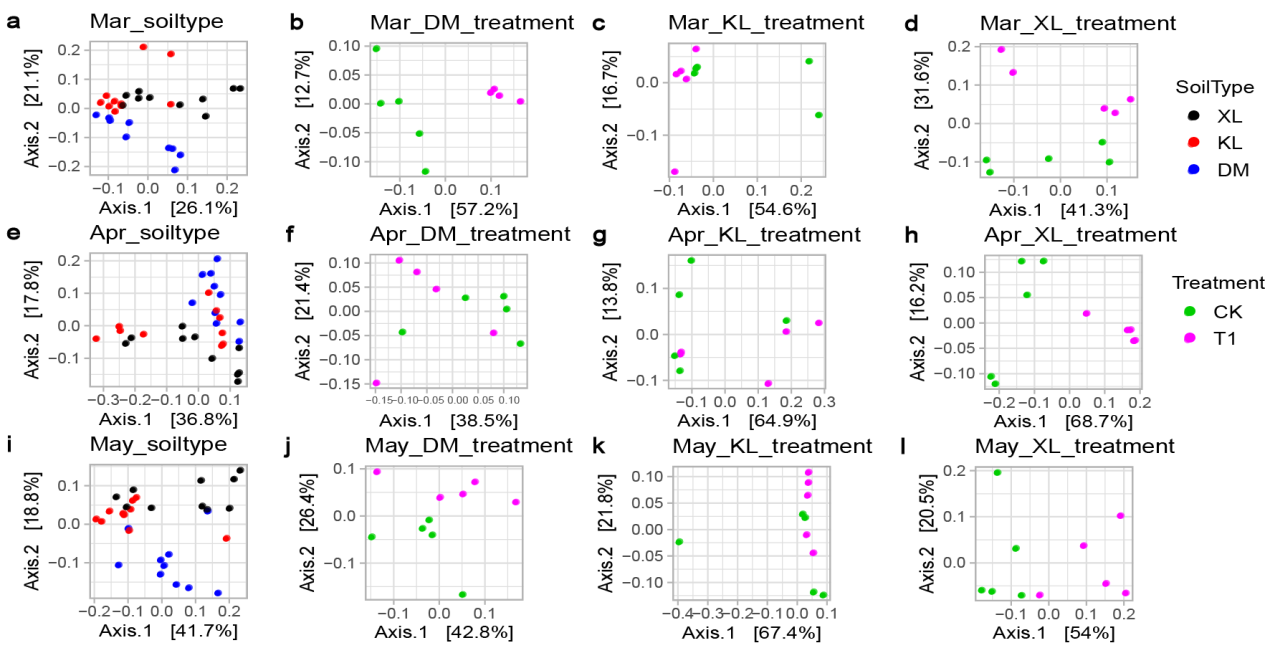


**Supplementary Fig. S2 PCoA of fungal microbiota using weighted UniFrac matrice for soil type factor and treatment factor. a,e,i** PCoA for soil type factor in different growth periods. **b–d** PCoA for treatment factor in different soil types in Mar. **f–h** PCoA for treatment factor in different soil types in Apr. **j–l** PCoA for treatment factor in different soil types in May. Mar, resume growth period; Apr, bolting period; May, maturation period; XL, loam; KL, sandy loam; DM, sandy soil; CK, plots without microbial product; T1, plots with microbial product.

**
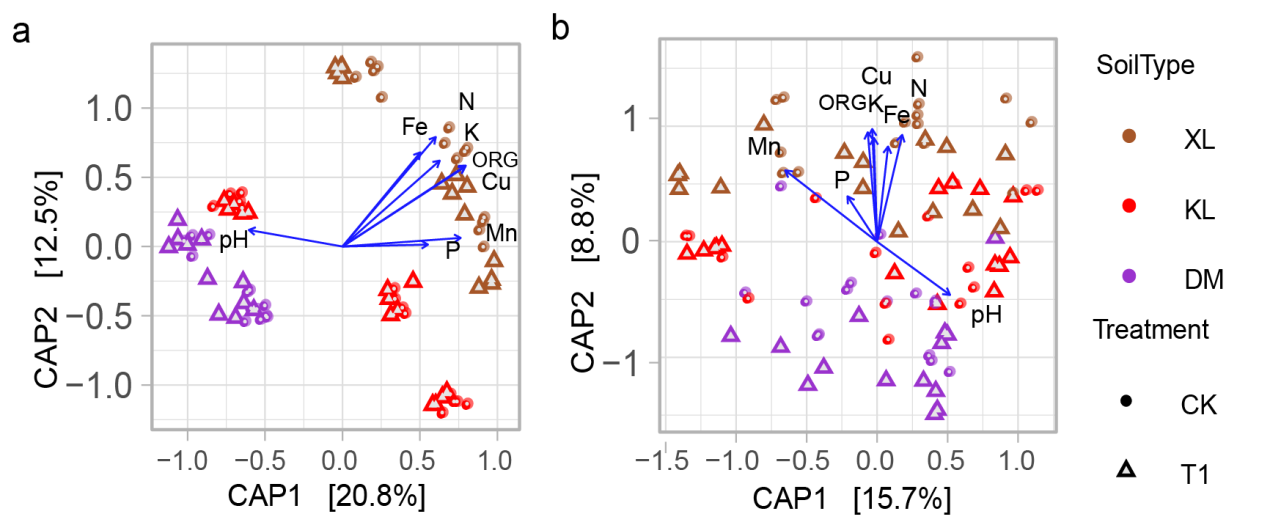
**

**Supplementary Fig. 3 CAP analysis based on Bray–Curtis distance for bacterial and fungal microbiota in whole dataset. a** The contribution of environmental factors of soil chemistry to differences in bacteria microbiota. **b** The contribution of environmental factors of soil chemistry to differences in fungal microbiota. XL, loam; KL, sandy loam; DM, sandy soil; CK, plots without microbial product; T1, plots with microbial product.


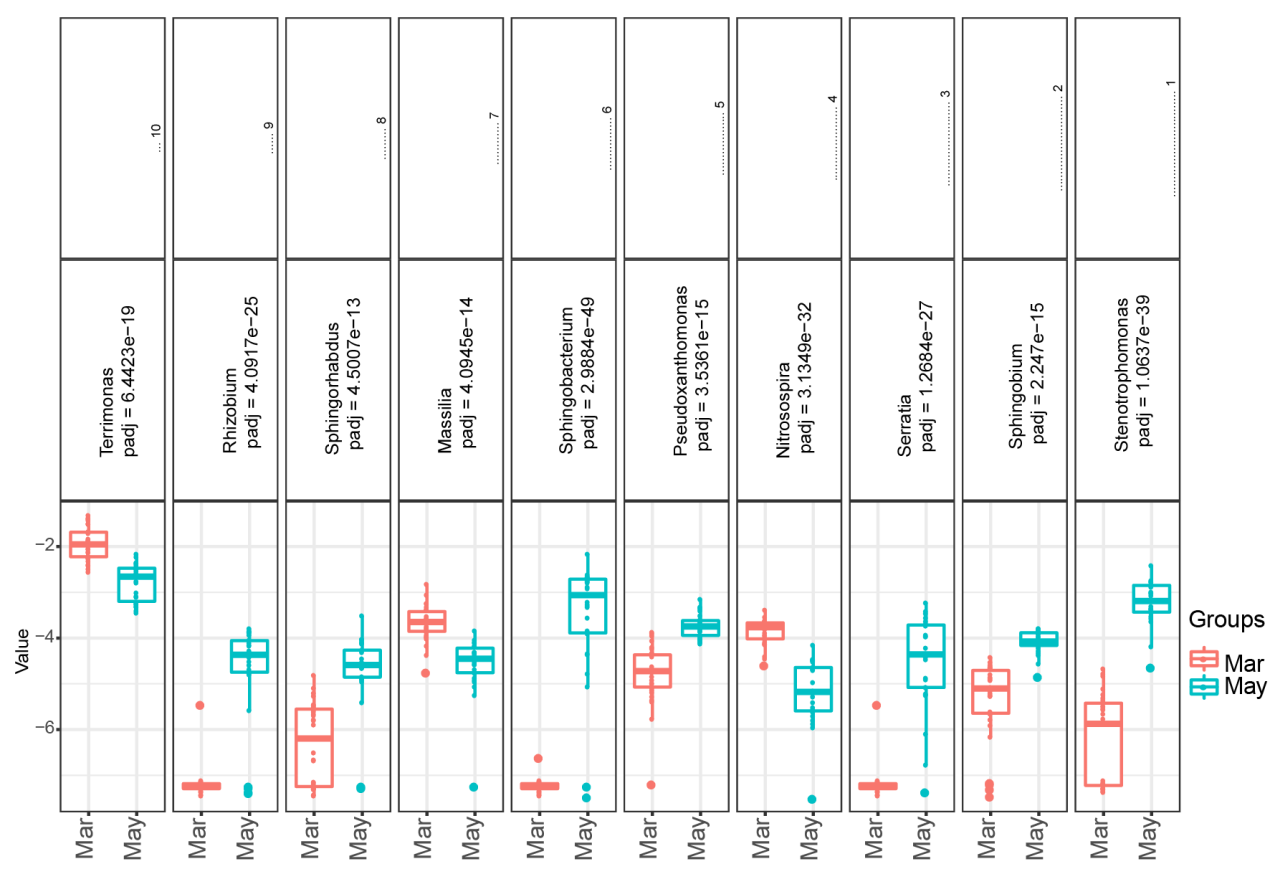


**Supplementary Fig. S4 Top10 differentially abundant genus of bacteria between Mar and May comparison groups in the whole datasets.** Corresponding adjusted *P*-values and rank of importance were detected by random forest classifier. Mar groups is red, May groups is blue. Mar, resume growth period; May, maturation period.


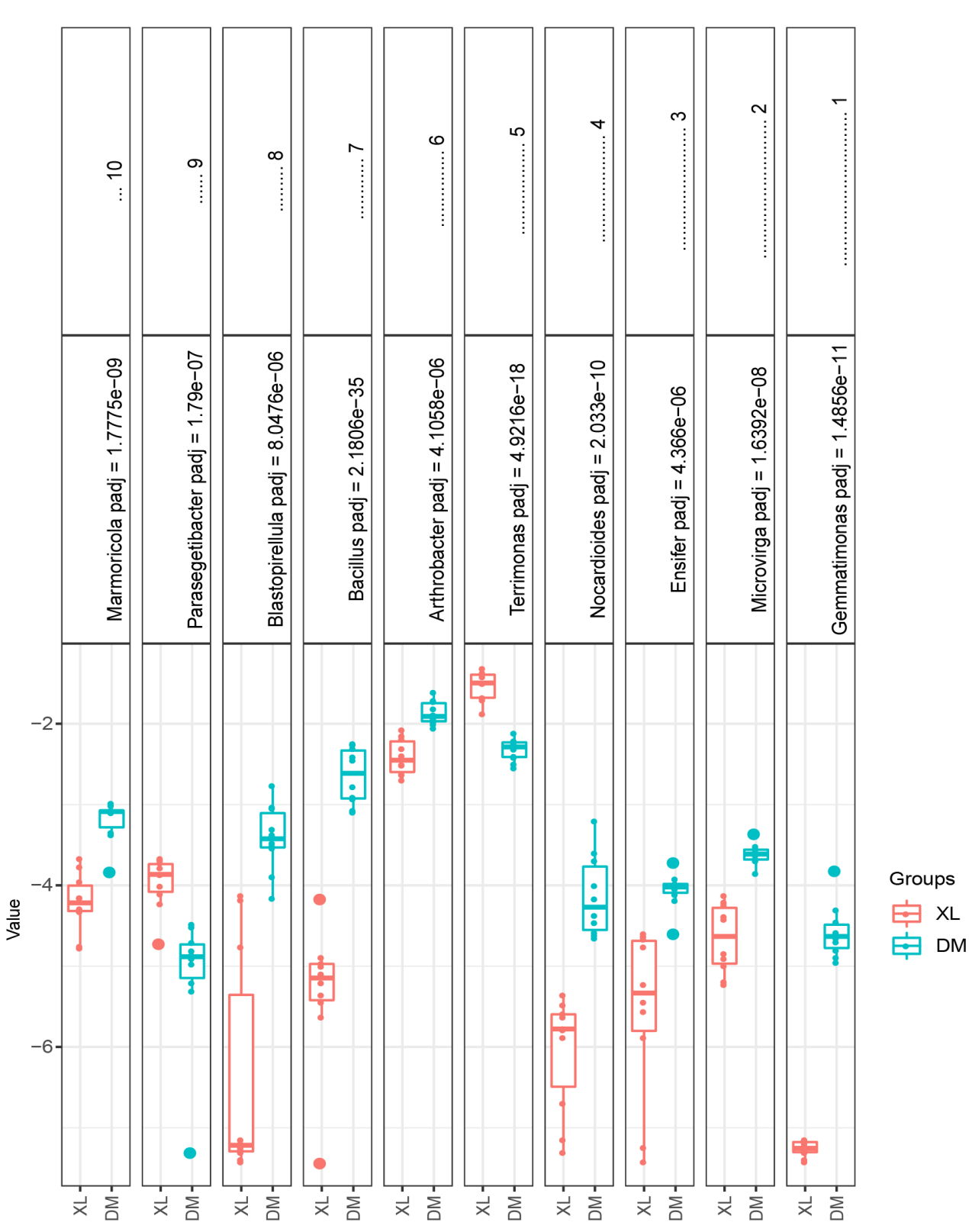


**Supplementary Fig. S5 Top10 differentially abundant genus of bacteria between XL and DM comparison groups in the Mar datasets.** Corresponding adjusted *P*-values and rank of importance were detected by random forest classifier. XL groups is red, DM groups is blue. Mar, resume growth period; XL, loam; DM, sandy soil.


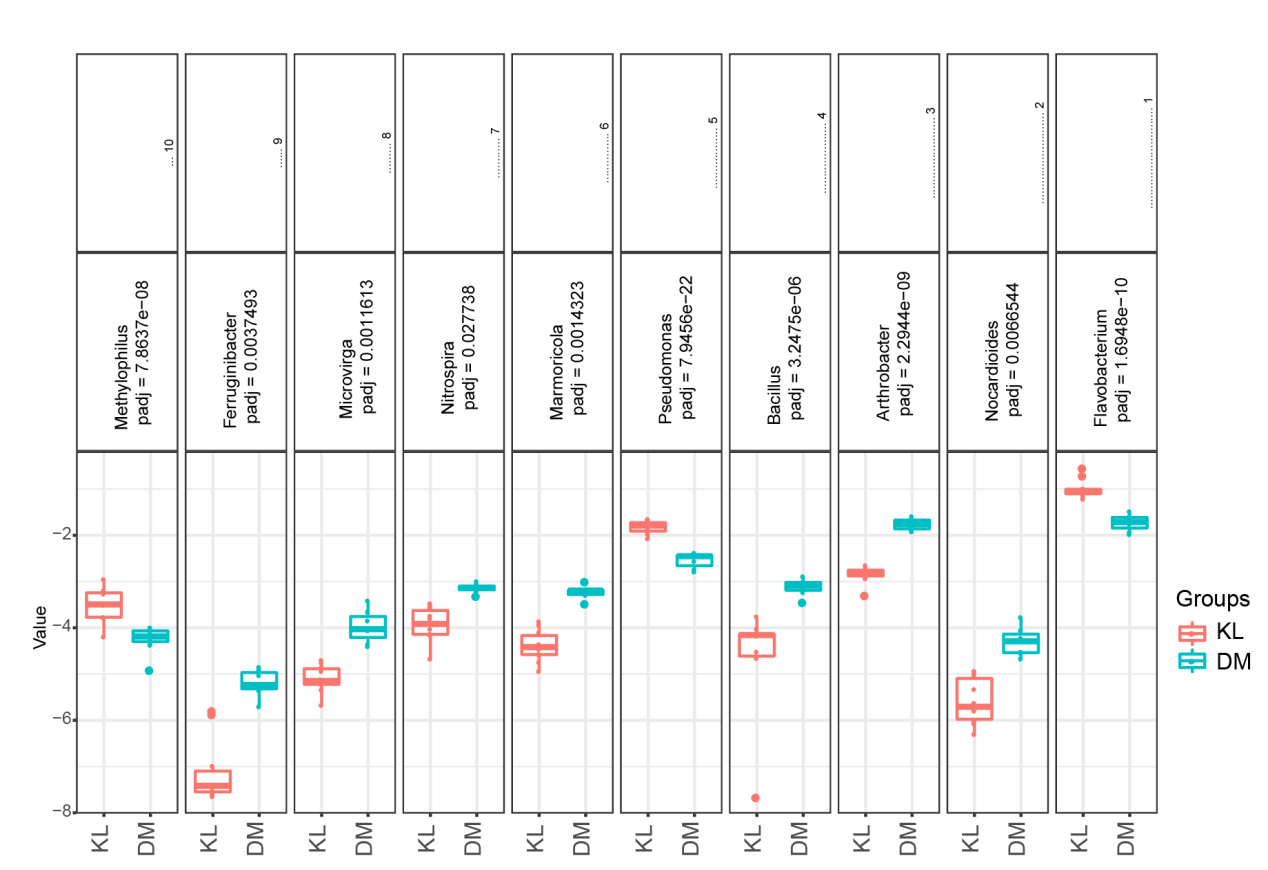


**Supplementary Fig. S6 Top10 differentially abundant genus of bacteria between KL and DM comparison groups in the Apr datasets.** Corresponding adjusted *P*-values and rank of importance were detected by random forest classifier. KL groups is red, DM groups is blue. Apr, bolting period; KL, sandy loam; DM, sandy soil.


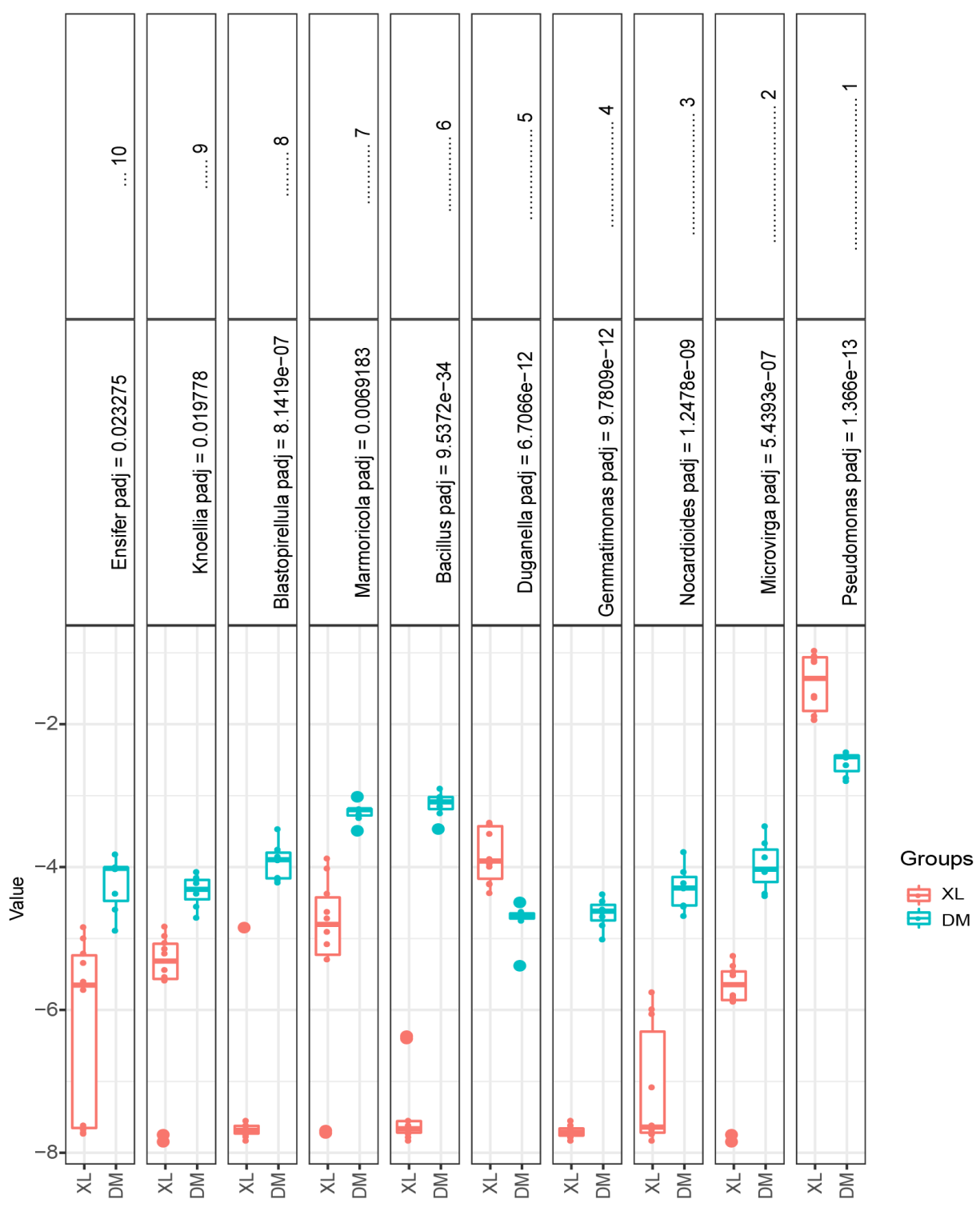


**Supplementary Fig. S7 Top10 differentially abundant genus of bacteria between XL and DM comparison groups in the Apr datasets.** Corresponding adjusted *P*-values and rank of importance were detected by random forest classifier. XL groups is red, DM groups is blue. Apr, bolting period; XL, loam; DM, sandy soil.


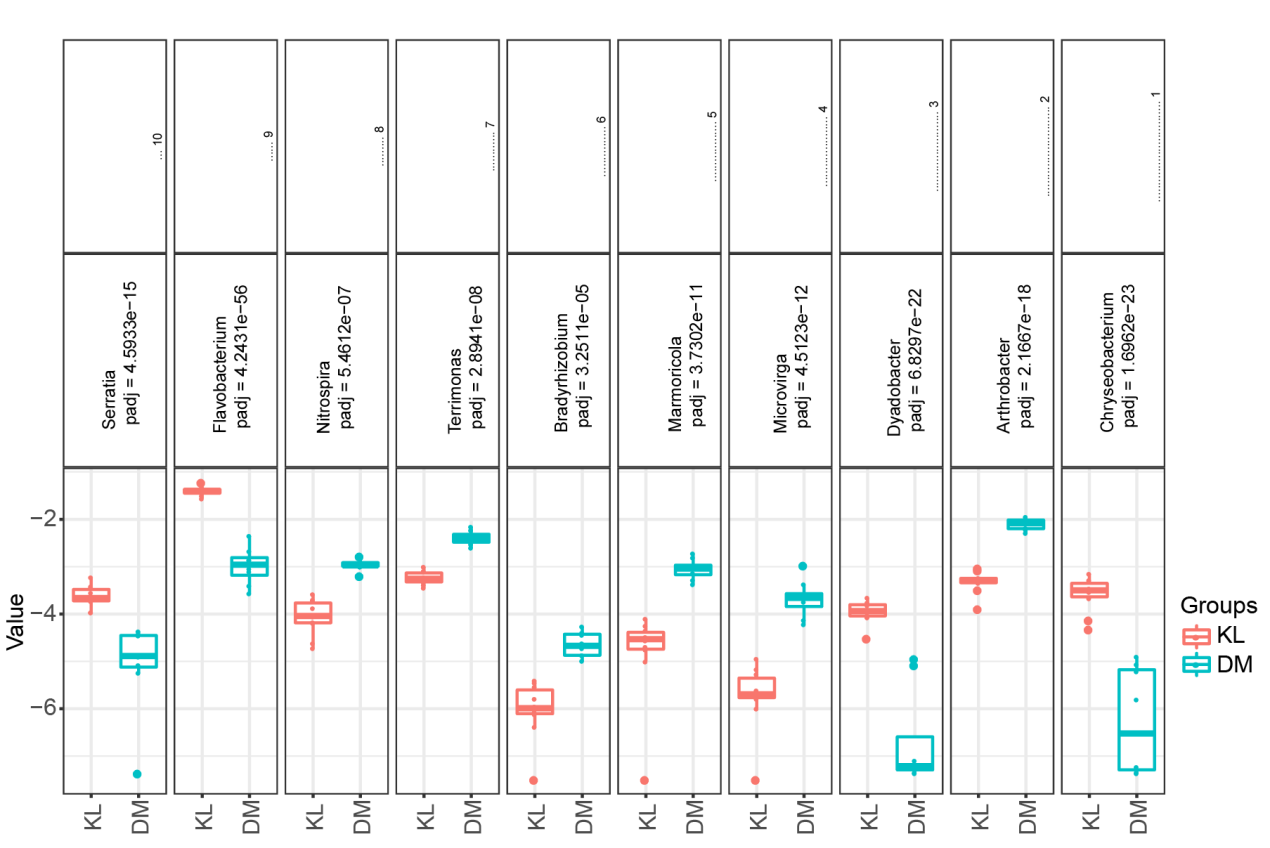


**Supplementary Fig. S8 Top10 differentially abundant genus of bacteria between KL and DM compared groups in the May datasets.** Corresponding adjusted *P*-values and rank of importance were detected by random forest classifier. KL groups is red, DM groups is blue. May, maturation period; KL, sandy loam; DM, sandy soil.


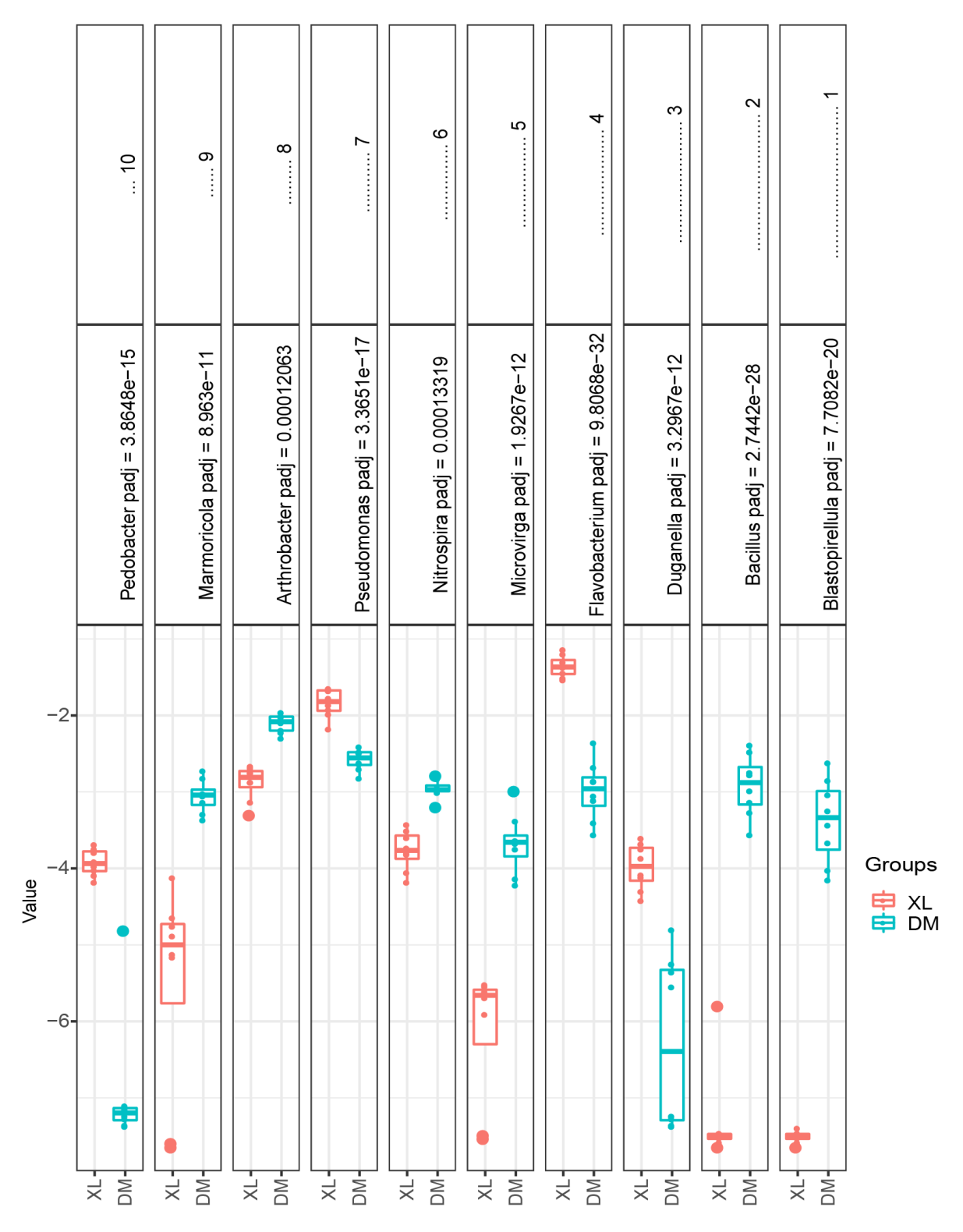


**Supplementary Fig. S9 Top10 differentially abundant genus of bacteria between XL and DM comparison groups in the May datasets.** Corresponding adjusted *P*-values and rank of importance were detected by random forest classifier. XL groups is red, DM groups is blue. May, maturation period; XL, loam; DM, sandy soil.


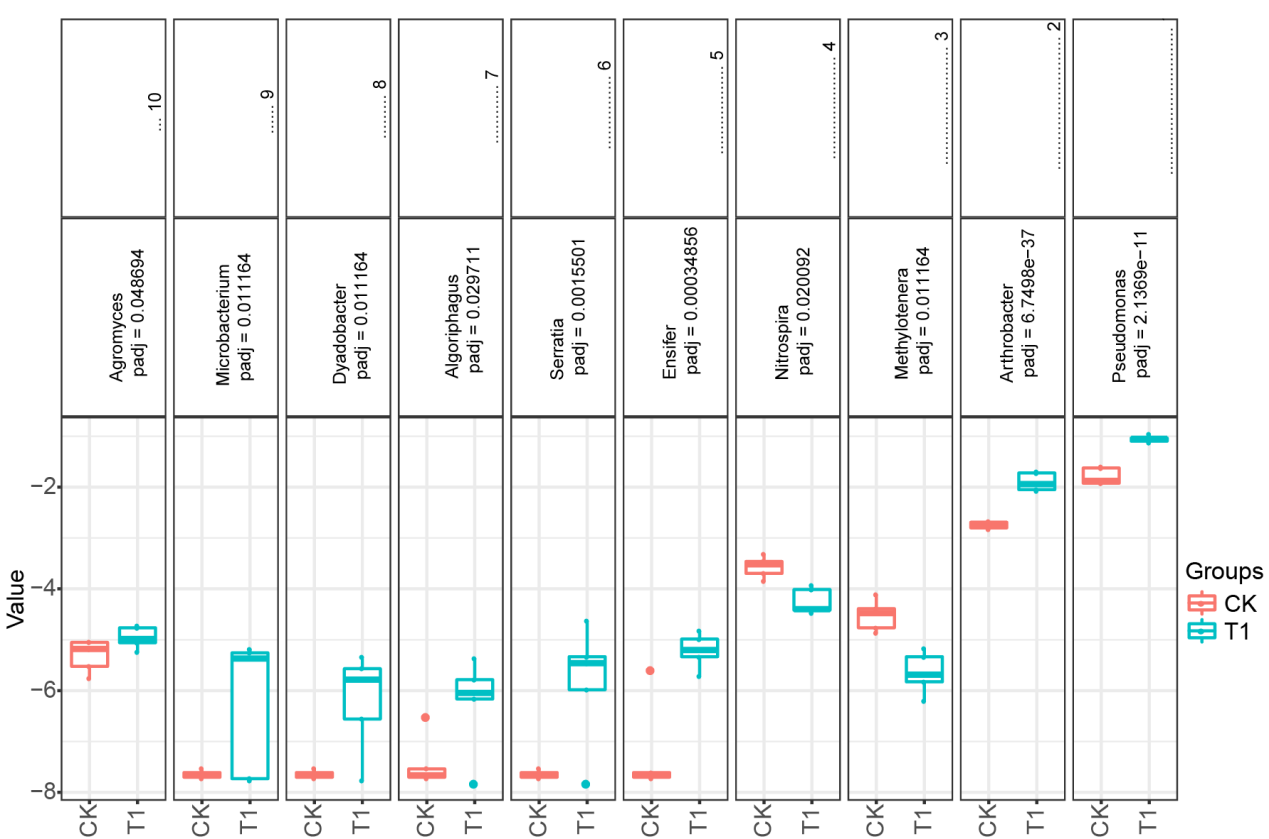


**Supplementary Fig. S10 Top10 differentially abundant genus of bacteria between T1 and CK comparison groups in the Apr–XL datasets.** Corresponding adjusted *P*-values and rank of importance were detected by random forest classifier. CK groups is red, T1 groups is blue. Apr, bolting period; XL, loam; CK, plots without microbial product; T1, plots with microbial product.


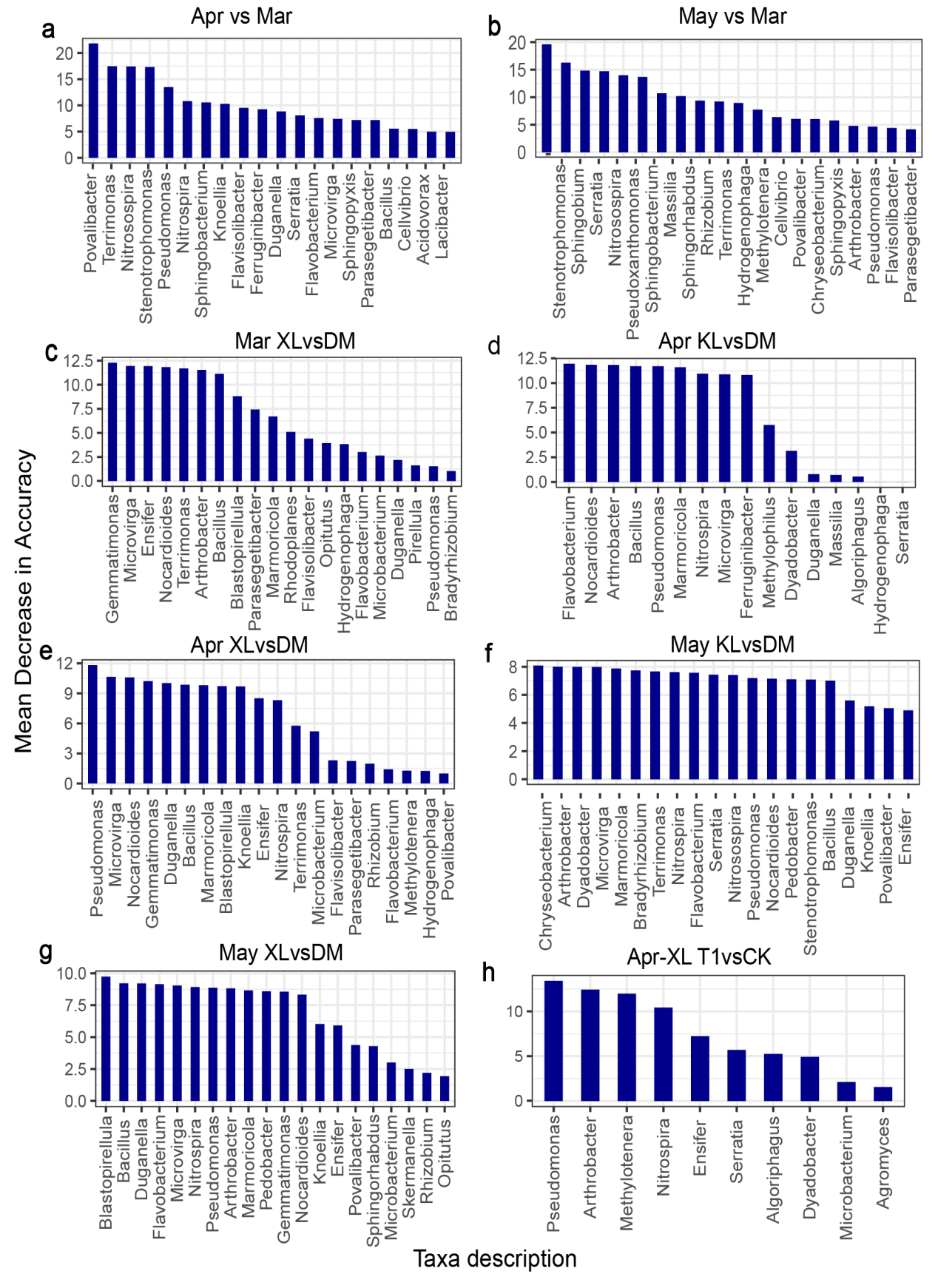


**Supplementary Fig. S11 Mean decrease accuracy values of differentially abundant genus which can provide a stand visual representation of important features obtained by random forest classifer in different comparison groups. a** Apr and Mar comparison groups in the whole datasets. **b** May and Mar comparison groups in the whole datasets. **c** XL and DM comparison groups in the Mar datasets. **d** KL and DM comparison groups in the Apr datasets. **e** KL and DM comparison groups in the Apr datasets. **f** KL and DM comparison groups in the May datasets. **g** XL and DM comparison groups in the May datasets. **h** T1 and CK comparison groups in the Apr-XL datasets. Mar, resume growth period; Apr, bolting period; May, maturation period; XL, loam; KL, sandy loam; DM, sandy soil; CK, plots without microbial product; T1, plots with microbial product.


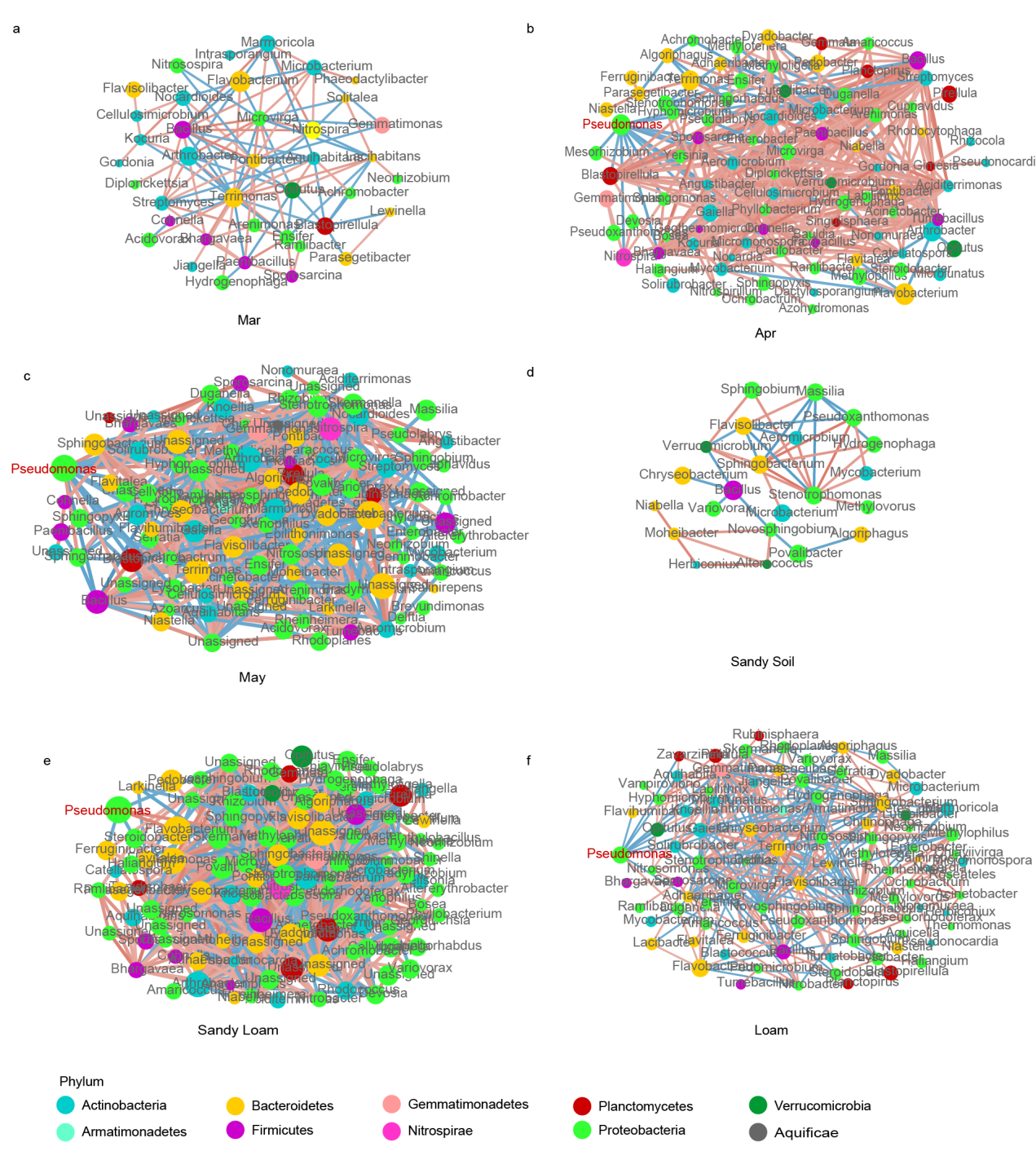


**Supplementary Fig. S12 Co-occurrence networks of Mar, Apr, May sandy soil, sandy loam and loam datasets.** Co-occurrence networks of Mar (**a**), Apr (**b**), May(**c**) sandy soil (**d**), sandy loam(**e**) and loam (**f**) datasets. Different colours of nodes represent different phylum of bacteria microbiota. Correlations between genus were expressed in different colors edges (positive correlation were represented as red edges, negative correlations were represented as blue edges), and the size of nodes indicated the abundance of genus. Mar, resume growth period; Apr, bolting period; May, maturation period.


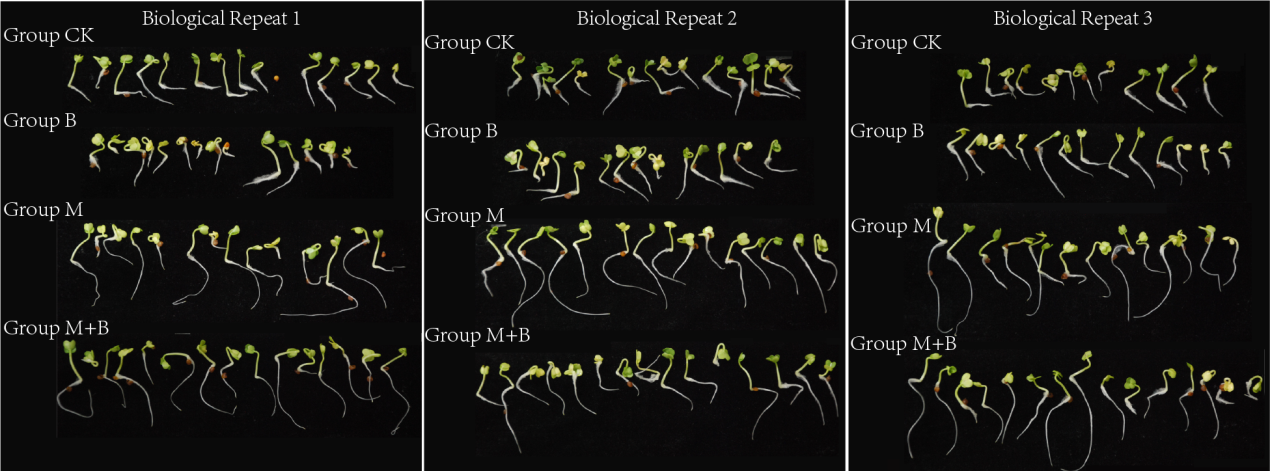


**Supplementary Fig. S13 SynCom promote radish seedlings growth.** Group CK used sterile water for the negative control. Group M consisted of six *Pseudomonas* sp. strains with different phylogenetic names mixed in equal proportions.Group M+B added B8-7 strain to M group in equal proportions. B group only contains B8-7 strain that was used to be the positive control.

**Supplementary Tables**

**Supplementary Table S1 Soil chemical situation in different growth periods, soil types and treatments.** Mar, resume growth period; Apr, bolting period; May, maturation period; XL, loam; KL, sandy loam; DM, sandy soil; CK, plots without microbial product; T1, plots with microbial product.

|  | pH | organic matter（g/kg） | N.tota l (g/kg) | P.available (mg/kg) | K.available (mg/kg) | Mn.available (mg/kg) | Fe.available (mg/kg) | Cu.available (mg/kg) |
| --- | --- | --- | --- | --- | --- | --- | --- | --- |
| Mar-XL-CK | 8.60 | 16.89 | 1.12 | 46.17 | 443.40 | 26.34 | 10.46 | 2.12 |
| Mar-XL-T1 | 8.44 | 17.96 | 1.22 | 65.39 | 520.28 | 24.04 | 9.14 | 2.07 |
| Mar-KL-CK | 8.11 | 12.36 | 0.83 | 32.55 | 367.01 | 19.04 | 7.61 | 1.15 |
| Mar-KL-T1 | 8.79 | 12.40 | 0.84 | 41.61 | 353.43 | 18.84 | 6.54 | 1.21 |
| Mar-DM-CK | 8.97 | 10.21 | 0.70 | 23.51 | 272.76 | 14.16 | 6.48 | 0.64 |
| Mar-DM-T1 | 8.83 | 10.07 | 0.74 | 23.13 | 265.22 | 13.64 | 6.46 | 0.64 |
| Apr-XL-CK | 8.47 | 19.25 | 1.15 | 15.94 | 464.73 | 23.41 | 9.87 | 2.39 |
| Apr-XL-T1 | 8.50 | 17.01 | 1.05 | 58.96 | 408.16 | 21.39 | 10.29 | 2.30 |
| Apr-KL-CK | 8.45 | 13.36 | 0.80 | 40.00 | 328.88 | 14.53 | 6.62 | 0.98 |
| Apr-KL-T1 | 8.42 | 14.01 | 0.85 | 64.73 | 315.16 | 19.76 | 9.84 | 1.79 |
| Apr-DM-CK | 8.68 | 11.52 | 0.66 | 22.46 | 322.32 | 14.54 | 5.87 | 0.63 |
| Apr-DM-T1 | 8.57 | 11.40 | 0.67 | 38.00 | 220.65 | 14.56 | 6.41 | 0.70 |
| May-XL-CK | 8.13 | 17.65 | 1.06 | 49.30 | 423.02 | 17.91 | 13.14 | 2.10 |
| May-XL-T1 | 7.93 | 17.22 | 1.02 | 54.67 | 538.78 | 25.43 | 15.75 | 2.19 |
| May-KL-CK | 8.47 | 12.34 | 0.74 | 41.72 | 354.70 | 15.22 | 9.23 | 1.13 |
| May-KL-T1 | 8.36 | 13.62 | 0.78 | 58.18 | 298.74 | 17.76 | 10.16 | 1.25 |
| May-DM-CK | 8.36 | 11.02 | 0.63 | 37.10 | 306.47 | 18.11 | 8.45 | 0.83 |
| May-DM-T1 | 8.49 | 12.23 | 0.69 | 31.11 | 213.16 | 16.36 | 11.05 | 0.78 |

**Supplementary Table S2 Tukey_HSD table of garlic yield.** XL, loam; KL, sandy loam; DM, sandy soil.

|  | diff | lwr | upr | *P* adj |
| --- | --- | --- | --- | --- |
| KL-CK--DM-CK | 1083.33333333333 | -3352.33614912157 | 5519.00281578823 | 0.95799894671465 |
| XL-CK--DM-CK | 4933.33333333333 | 497.663850878426 | 9369.00281578823 | 0.0264980639509778 |
| DM-T1--DM-CK | 6500 | 2064.33051754509 | 10935.6694824549 | 0.00366934498973726 |
| XL-T1--DM-CK | 8216.66666666666 | 3780.99718421176 | 12652.3361491216 | 0.000490089149308592 |
| KL-T1--DM-CK | 8950 | 4514.33051754509 | 13385.6694824549 | 0.000220584300203108 |
| XL-CK--KL-CK | 3850 | -585.669482454902 | 8285.6694824549 | 0.103859499709874 |
| DM-T1--KL-CK | 5416.66666666667 | 980.997184211766 | 9852.33614912157 | 0.0142825798111564 |
| XL-T1--KL-CK | 7133.33333333333 | 2697.66385087843 | 11569.0028157882 | 0.00170661340560707 |
| KL-T1--KL-CK | 7866.66666666667 | 3430.99718421177 | 12302.3361491216 | 0.000726922955477716 |
| DM-T1--XL-CK | 1566.66666666667 | -2869.00281578823 | 6002.33614912157 | 0.835123220129507 |
| XL-T1--XL-CK | 3283.33333333333 | -1152.33614912157 | 7719.00281578823 | 0.20256676078689 |
| KL-T1--XL-CK | 4016.66666666667 | -419.002815788234 | 8452.33614912157 | 0.0846033384284177 |
| XL-T1--DM-T1 | 1716.66666666666 | -2719.00281578824 | 6152.33614912157 | 0.779929312517426 |
| KL-T1--DM-T1 | 2450 | -1985.6694824549 | 6885.6694824549 | 0.470036730701255 |
| KL-T1--XL-T1 | 733.333333333336 | -3702.33614912157 | 5169.00281578824 | 0.992249913533519 |

**Supplementary Table S3 Dunnetts test table of garlic bulb diameter.** Dunn's test of multiple comparisons using rank sums : holm. XL, loam; KL, sandy loam; DM, sandy soil; CK, plots without microbial product; T1, plots with microbial product.

|  | mean.rank.diff | pval |  |
| --- | --- | --- | --- |
| DM-T1--DM-CK | 119.670 | 1.1e-05 | *** |
| KL-CK--DM-CK | 88.425 | 0.0028 | ** |
| KL-T1--DM-CK | 166.025 | 1.6e-10 | *** |
| XL-CK--DM-CK | 80.120 | 0.0065 | ** |
| XL-T1--DM-CK | 251.990 | <2e-16 | *** |
| KL-CK--DM-T1 | -31.245 | 0.4049 |  |
| KL-T1--DM-T1 | 46.355 | 0.2345 |  |
| XL-CK--DM-T1 | -39.550 | 0.3200 |  |
| XL-T1--DM-T1 | 132.320 | 7.4e-07 | *** |
| KL-T1--KL-CK | 77.600 | 0.0077 | ** |
| XL-CK--KL-CK | -8.305 | 0.7348 |  |
| XL-T1--KL-CK | 163.565 | 3.0e-10 | *** |
| XL-CK--KL-T1 | -85.905 | 0.0036 | ** |
| XL-T1--KL-T1 | 85.965 | 0.0036 | ** |
| XL-T1--XL-CK | 171.870 | 3.3e-11 | *** |
| Signif. codes: 0, '***': 0.001, '**': 0.01, '*': 0.05 , '.' 0.1, ' ': 1. | | | |

**Supplementary Table S4 Dunnetts test table of radish seedlings length.** Dunn's test of multiple comparisons using rank sums : holm. Group CK used sterile water for the negative control. Group M consisted of six Pseudomonas sp. strains with different phylogenetic names mixed in equal proportions.Group M+B added B8-7 strain to M group in equal proportions. B group only contains B8-7 strain that was used to be the positive control.

|  | mean.rank.diff | pval |  |
| --- | --- | --- | --- |
| B--CK | 4.190244 | 0.6983 |  |
| M--CK | 67.175958 | 1.9e-09 | *** |
| M+B--CK | 56.368022 | 3.2e-07 | *** |
| M--B | 62.985714 | 2.3e-08 | *** |
| M+B--B | 52.177778 | 2.4e-06 | *** |
| M+B--M | -10.807937 | 0.6007 |  |

Signif. codes: 0, '***': 0.001, '**': 0.01, '*': 0.05 , '.' 0.1, ' ': 1.

**Supplementary Table S5 Taxonomy of bacterial isolates that make up the synthetic community.** Phylogenetic names of all 263 isolates which isolated from garlic rhizosphere soil was obtained by blast against NCBI comparison. Six Pseudomonas strains with different phylogenetic names and a Bacillus strain were selected to compose different synthetic community groups.

| Strain ID | Phylum | Class | Family | Genus | Phylogenetic names |
| --- | --- | --- | --- | --- | --- |
| P3-7 | Proteobacteria | Gammaproteobacteria | Pseudomonadaceae | Pseudomonas | cedrina |
| P3-22 | Proteobacteria | Gammaproteobacteria | Pseudomonadaceae | Pseudomonas | baetica |
| P6-3 | Proteobacteria | Gammaproteobacteria | Pseudomonadaceae | Pseudomonas | migulae |
| P6-33 | Proteobacteria | Gammaproteobacteria | Pseudomonadaceae | Pseudomonas | fluorescens |
| P7-5 | Proteobacteria | Gammaproteobacteria | Pseudomonadaceae | Pseudomonas | reinekei |
| P7-14 | Proteobacteria | Gammaproteobacteria | Pseudomonadaceae | Pseudomonas | frederiksbergensis |
| B8-7 | Firmicutes | Bacilli | Bacillaceae | Bacillus | simplex |

**Supplementary Table S6 16S rRNA gene sequence of strains in SynComs**

| strains | 16S rRNA gene sequence |
| --- | --- |
| P3-7 | 5’-GCTTCTCTTGAGAGCGGCGGACGGGTGAGTAAAGCCTAGGAATCTGCCTGGTAGTGGGGGATAACGTTCGGAAACGGACGCTAATACCGCATACGTCCTACGGGAGAAAGCAGGGGACCTTCGGGCCTTGCGCTATCAGATGAGCCTAGGTCGGATTAGCTAGTTGGTGAGGTAATGGCTCACCAAGGCGACGATCCGTAACTGGTCTGAGAGGATGATCAGTCACACTGGAACTGAGACACGGTCCAGACTCCTACGGGAGGCAGCAGTGGGGAATATTGGACAATGGGCGAAAGCCTGATCCAGCCATGCCGCGTGTGTGAAGAAGGTCTTCGGATTGTAAAGCACTTTAAGTTGGGAGGAAGGGTTGTAGATTAATACTCTGCAATTTTGACGTTACCGACAGAATAAGCACCGGCTAACTCTGTGCCAGCAGCCGCGGTAATACAGAGGGTGCAAGCGTTAATCGGAATTACTGGGCGTAAAGCGCGCGTAGGTGGTTTGTTAAGTTGGATGTGAAATCCCCGGGCTCAACCTGGGAACTGCATTCAAAACTGACTGACTAGAGTGTGGTAGAGGGTGGTGGAATTTCCTGTGTAGCGGTGAAATGCGTAGATATAGGAAGGAACACCAGTGGCGAAGGCGACCACCTGGACCAACACTGACACTGAGGTGCGAAAGCGTGGGGAGCAAACAGGATTAGATACCCTGGTAGTCCACGCCGTAAACGATGTCAACTAGCCGTTGGGAGCCTTGAGCTCTTAGTGGCGCAGCTAACGCATTAAGTTGACCGCCTGGGGAGTACGGCCGCAAGGTTAAAACTCAAATGAATTGACGGGGGCCCGCACAAGCGGTGGAGCATGTGGTTTAATTCGAAGCAACGCGAAGAACCTTACCAGGCCTTGACATCCAATGAACTTTCTAGAGATAGATTGGTGCCTTCGGGAACATTGAGACAGGTGCTGCATGGCTGTCGTCAGCTCGTGTCGTGAGATGTTGGGTTAAGTCCCGTAACGAGCGCAACCCTTGTCCTTAGTTACCAGCACGTTATGGTGGGCACTCTAAGGAGACTGCCGGTGACAAACCGGAGGAAGGTGGGGATGACGTCAAGTCATCATGGCCCTTACGGCCTGGGCTACACACGTGCTACAATGGTCGGTACAGAGGGTTGCCAAGCCGCGAGGTGGAGCTAATCCCACAAAACCGATCGTAGTCCGGATCGCAGTCTGCAACTCGACTGCGTGAAGTCGGAATCGCTAGTATCGC-3’ |
| P3-22 | 5’-ATCTGCCTGGTAGTGGGGGACAACGTTTCGAAAGGAACGCTAATACCGCATACGTCCTACGGGAGAAAGCAGGGGACCTTCGGGCCTTGCGCTATCAGATGAGCCTAGGTCGGATTAGCTAGTTGGTGAGGTAATGGCTCACCAAGGCGACGATCCGTAACTGGTCTGAGAGGATGATCAGTCACACTGGAACTGAGACACGGTCCAGACTCCTACGGGAGGCAGCAGTGGGGAATATTGGACAATGGGCGAAAGCCTGATCCAGCCATGCCGCGTGTGTGAAGAAGGTCTTCGGATTGTAAAGCACTTTAAGTTGGGAGGAAGGGCAGTAAATTAATACTTTGCTGTTTTGACGTTACCGACAGAATAAGCACCGGCTAACTCTGTGCCAGCAGCCGCGGTAATACAGAGGGTGCAAGCGTTAATCGGAATTACTGGGCGTAAAGCGCGCGTAGGTGGTTTGTTAAGTTGGATGTGAAATCCCCGGGCTCAACCTGGGAACTGCATCCAAAACTGGCAAGCTAGAGTATGGTAGAGGGTGGTGGAATTTCCTGTGTAGCGGTGAAATGCGTAGATATAGGAAGGAACACCAGTGGCGAAGGCGACCACCTGGACTGATACTGACACTGAGGTGCGAAAGCGTGGGGAGCAAACAGGATTAGATACCCTGGTAGTCCACGCCGTAAACGATGTCAACTAGCCGTTGGGAGCCTTGAGCTCTTAGTGGCGCAGCTAACGCATTAAGTTGACCGCCTGGGGAGTACGGCCGCAAGGTTAAAACTCAAATGAATTGACGGGGGCCCGCACAAGCGGTGGAGCATGTGGTTTAATTCGAAGCAACGCGAAGAACCTTACCAGGCCTTGACATCCAATGAACTTTCCAGAGATGGATTGGTGCCTTCGGGAACATTGAGACAGGTGCTGCATGGCTGTCGTCAGCTCGTGTCGTGAGATGTTGGGTTAAGTCCCGTAACGAGCGCAACCCTTGTCCTTAGTTACCAGCACGTAATGGTGGGCACTCTAAGGAGACTGCCGGTGACAAACCGGAGGAAGGTGGGGATGACGTCAAGTCATCATGGCCCTTACGGCCTGGGCTACACACGTGCTACAATGGTCGGTACAAAGGGTTGCCAAGCCGCGAGGTGGAGCTAATCCCATAAAACCGATCGTAGTCCGGATCGCAGTCTGCAACTCGACTGCGTGAAGTCGGAATCGCTAGTAATCGCGAAT-3’ |
| P6-3 | 5’-CTTGCTTCTCTTGAGAGCGGCGGACGGGTGAGTAATGCCTAGGAATCTGCCTGGTAGTGGGGGATAACGCTCGGAAACGGACGCTAATACCGCATACGTCCTACGGGAGAAAGCAGGGGACCTTCGGGCCTTGCGCTATCAGATGAGCCTAGGTCGGATTAGCTAGTTGGTGAGGTAATGGCTCACCAAGGCGACGATCCGTAACTGGTCTGAGAGGATGATCAGTCACACTGGAACTGAGACACGGTCCAGACTCCTACGGGAGGCAGCAGTGGGGAATATTGGACAATGGGCGAAAGCCTGATCCAGCCATGCCGCGTGTGTGAAGAAGGTCTTCGGATTGTAAAGCACTTTAAGTTGGGAGGAAGGGCAGTAAATTAATACTTTGCTGTTTTGACGTTACCGACAGAATAAGCACCGGCTAACTCTGTGCCAGCAGCCGCGGTAATACAGAGGGTGCAAGCGTTAATCGGAATTACTGGGCGTAAAGCGCGCGTAGGTGGTTTGTTAAGTTGGATGTGAAATCCCCGGGCTCAACCTGGGAACTGCATTCAAAACTGACAAGCTAGAGTATGGTAGAGGGTGGTGGAATTTCCTGTGTAGCGGTGAAATGCGTAGATATAGGAAGGAACACCAGTGGCGAAGGCGACCACCTGGACTGATACTGACACTGAGGTGCGAAAGCGTGGGGAGCAAACAGGATTAGATACCCTGGTAGTCCACGCCGTAAACGATGTCAACTAGCCGTTGGGAGCCTTGAGCTCTTAGTGGCGCAGCTAACGCATTAAGTTGACCGCCTGGGGAGTACGGCCGCAAGGTTAAAACTCAAATGAATTGACGGGGGCCCGCACAAGCGGTGGAGCATGTGGTTTAATTCGAAGCAACGCGAAGAACCTTACCAGGCCTTGACATCCAATGAACTTTCCAGAGATGGATTGGTGCCTTCGGGAACATTGAGACAGGTGCTGCATGGCTGTCGTCAGCTCGTGTCGTGAGATGTTGGGTTAAGTCCCGTAACGAGCGCAACCCTTGTCCTTAGTTACCAGCACGTAATGGTGGGCACTCTAAGGAGACTGCCGGTGACAAACCGGAGGAAGGTGGGGATGACGTCAAGTCATCATGGCCCTTACGGCCTGGGCTACACACGTGCTACAATGGTCGGTACAGAGGGTTGCCAAGCCGCGAGGTGGAGCTAATCCCAGAAAACCGATCGTAGTCCGGATCGCAGTCTGCAACTCGACTGCGTGAAGTCGGAATCGCTAGTAA-3’ |
| P6-33 | 5’-GTGAGTAATGCCTAGGAATCTGCCTGGTAGTGGGGGACAACGTTTCGAAAGGAACGCTAATACCGCATACGTCCTACGGGAGAAAGCAGGGGACCTTCGGGCCTTGCGCTATCAGATGAGCCTAGGTCGGATTAGCTAGTTGGTGAGGTAATGGCTCACCAAGGCGACGATCCGTAACTGGTCTGAGAGGATGATCAGTCACACTGGAACTGAGACACGGTCCAGACTCCTACGGGAGGCAGCAGTGGGGAATATTGGACAATGGGCGAAAGCCTGATCCAGCCATGCCGCGTGTGTGAAGAAGGTCTTCGGATTGTAAAGCACTTTAAGTTGGGAGGAAGGGTTGTAGATTAATACTCTGCAATTTTGACGTTACCGACAGAATAAGCACCGGCTAACTCTGTGCCAGCAGCCGCGGTAATACAGAGGGTGCAAGCGTTAATCGGAATTACTGGGCGTAAAGCGCGCGTAGGTGGTTTGTTAAGTTGGATGTGAAAGCCCCGGGCTCAACCTGGGAACTGCATTCAAAACTGACAAGCTAGAGTATGGTAGAGGGTGGTGGAATTTCCTGTGTAGCGGTGAAATGCGTAGATATAGGAAGGAACACCAGTGGCGAAGGCGACCACCTGGACTGATACTGACACTGAGGTGCGAAAGCGTGGGGAGCAAACAGGATTAGATACCCTGGTAGTCCACGCCGTAAACGATGTCAACTAGCCGTTGGGAGCCTTGAGCTCTTAGTGGCGCAGCTAACGCATTAAGTTGACCGCCTGGGGAGTACGGCCGCAAGGTTAAAACTCAAATGAATTGACGGGGGCCCGCACAAGCGGTGGAGCATGTGGTTTAATTCGAAGCAACGCGAAGAACCTTACCAGGCCTTGACATCCAATGAACTTTCCAGAGATGGATTGGTGCCTTCGGGAACATTGAGACAGGTGCTGCATGGCTGTCGTCAGCTCGTGTCGTGAGATGTTGGGTTAAGTCCCGTAACGAGCGCAACCCTTGTCCTTAGTTACCAGCACGTAATGGTGGGCACTCTAAGGAGACTGCCGGTGACAAACCGGAGGAAGGTGGGGATGACGTCAAGTCATCATGGCCCTTACGGCCTGGGCTACACACGTGCTACAATGGTCGGTACAGAGGGTTGCCAAGCCGCGAGGTGGAGCTAATCCCACAAAACCGATCGTAGTCCGGATCGCAGTCTGCAACTCGACTGCGTGAAGTCGGAATCGCTA-3’ |
| P7-5 | 5’-GCGGCGGACGGGTGAGTAATGCCTAGGAATCTGCCTGGTAGTGGGGGACAACGTTTCGAAAGGAACGCTAATACCGCATACGTCCTACGGGAGAAAGCAGGGGACCTTCGGGCCTTGCGCTATCAGATGAGCCTAGGTCGGATTAGCTAGTTGGTGAGGTAATGGCTCACCAAGGCGACGATCCGTAACTGGTCTGAGAGGATGATCAGTCACACTGGAACTGAGACACGGTCCAGACTCCTACGGGAGGCAGCAGTGGGGAATATTGGACAATGGGCGAAAGCCTGATCCAGCCATGCCGCGTGTGTGAAGAAGGTCTTCGGATTGTAAAGCACTTTAAGTTGGGAGGAAGGGTTGTAGATTAATACTCTGCAATTTTGACGTTACCGACAGAATAAGCACCGGCTAACTCTGTGCCAGCAGCCGCGGTAATACAGAGGGTGCAAGCGTTAATCGGAATTACTGGGCGTAAAGCGCGCGTAGGTGGTTCGTTAAGTTGGATGTGAAATCCCCGGGCTCAACCTGGGAACTGCATTCAAAACTGTCGAGCTAGAGTATGGTAGAGGGTGGTGGAATTTCCTGTGTAGCGGTGAAATGCGTAGATATAGGAAGGAACACCAGTGGCGAAGGCGACCACCTGGACTGATACTGACACTGAGGTGCGAAAGCGTGGGGAGCAAACAGGATTAGATACCCTGGTAGTCCACGCCGTAAACGATGTCAACTAGCCGTTGGGAGCCTTGAGCTCTTAGTGGCGCAGCTAACGCATTAAGTTGACCGCCTGGGGAGTACGGCCGCAAGGTTAAAACTCAAATGAATTGACGGGGGCCCGCACAAGCGGTGGAGCATGTGGTTTAATTCGAAGCAACGCGAAGAACCTTACCAGGCCTTGACATCCAATGAACTTTCCAGAGATGGATTGGTGCCTTCGGGAACATTGAGACAGGTGCTGCATGGCTGTCGTCAGCTCGTGTCGTGAGATGTTGGGTTAAGTCCCGTAACGAGCGCAACCCTTGTCCTTAGTTACCAGCACGTTATGGTGGGCACTCTAAGGAGACTGCCGGTGACAAACCGGAGGAAGGTGGGGATGACGTCAAGTCATCATGGCCCTTACGGCCTGGGCTACACACGTGCTACAATGGTCGGTACAAAGGGTTGCCAAGCCGCGAGGTGGAGCTAATCCCATAAAACCGATCGTAGTCCGGATCGCAGTCTGCAACTCGACTGCGTGAAGTCGGAATCGCTAGTAA-3’ |
| P7-14 | 5’-TGTACCTGGTGGCGAGCGGCGGACGGGTGAGTAATGCCTAGGAATCTGCCTGGTAGTGGGGGATAACGCTCGGAAACGGACGCTAATACCGCATACGTCCTACGGGAGAAAGCAGGGGACCTTCGGGCCTTGCGCTATCAGATGAGCCTAGGTCGGATTAGCTAGTTGGTGAGGTAATGGCTCACCAAGGCGACGATCCGTAACTGGTCTGAGAGGATGATCAGTCACACTGGAACTGAGACACGGTCCAGACTCCTACGGGAGGCAGCAGTGGGGAATATTGGACAATGGGCGAAAGCCTGATCCAGCCATGCCGCGTGTGTGAAGAAGGTCTTCGGATTGTAAAGCACTTTAAGTTGGGAGGAAGGGCAGTTACCTAATACGTAATTGTTTTGACGTTACCGACAGAATAAGCACCGGCTAACTCTGTGCCAGCAGCCGCGGTAATACAGAGGGTGCAAGCGTTAATCGGAATTACTGGGCGTAAAGCGCGCGTAGGTGGTTCGTTAAGTTGGATGTGAAATCCCCGGGCTCAACCTGGGAACTGCATTCAAAACTGACGAGCTAGAGTATGGTAGAGGGTGGTGGAATTTCCTGTGTAGCGGTGAAATGCGTAGATATAGGAAGGAACACCAGTGGCGAAGGCGACCACCTGGACTGATACTGACACTGAGGTGCGAAAGCGTGGGGAGCAAACAGGATTAGATACCCTGGTAGTCCACGCCGTAAACGATGTCAACTAGCCGTTGGGAGCCTTGAGCTCTTAGTGGCGCAGCTAACGCATTAAGTTGACCGCCTGGGGAGTACGGCCGCAAGGTTAAAACTCAAATGAATTGACGGGGGCCCGCACAAGCGGTGGAGCATGTGGTTTAATTCGAAGCAACGCGAAGAACCTTACCAGGCCTTGACATCCAATGAACTTTCCAGAGATGGATTGGTGCCTTCGGGAACATTGAGACAGGTGCTGCATGGCTGTCGTCAGCTCGTGTCGTGAGATGTTGGGTTAAGTCCCGTAACGAGCGCAACCCTTGTCCTTAGTTACCAGCACGTAATGGTGGGCACTCTAAGGAGACTGCCGGTGACAAACCGGAGGAAGGTGGGGATGACGTCAAGTCATCATGGCCCTTACGGCCTGGGCTACACACGTGCTACAATGGTCGGTACAGAGGGTTGCCAAGCCGCGAGGTGGAGCTAATCCCAGAAAACCGATCGTAGTCCGGATCGCAGTCTGCAACTCGACTGCGTGAAGTCGGAATCGCTA-3’ |
| B8-7 | 5’-GTGGGCAACCTGCCTATAAGACTGGGATAACTTCGGGAAACCGGAGCTAATACCGGATACGTTCTTTTCTCGCATGAGAGAAGATGGAAAGACGGTTTACGCTGTCACTTATAGATGGGCCCGCGGCGCATTAGCTAGTTGGTGAGGTAATGGCTCACCAAGGCGACGATGCGTAGCCGACCTGAGAGGGTGATCGGCCACACTGGGACTGAGACACGGCCCAGACTCCTACGGGAGGCAGCAGTAGGGAATCTTCCGCAATGGACGAAAGTCTGACGGAGCAACGCCGCGTGAACGAAGAAGGCCTTCGGGTCGTAAAGTTCTGTTGTTAGGGAAGAACAAGTACCAGAGTAACTGCTGGTACCTTGACGGTACCTAACCAGAAAGCCACGGCTAACTACGTGCCAGCAGCCGCGGTAATACGTAGGTGGCAAGCGTTGTCCGGAATTATTGGGCGTAAAGCGCGCGCAGGTGGTTCCTTAAGTCTGATGTGAAAGCCCACGGCTCAACCGTGGAGGGTCATTGGAAACTGGGGAACTTGAGTGCAGAAGAGGAAAGTGGAATTCCAAGTGTAGCGGTGAAATGCGTAGAGATTTGGAGGAACACCAGTGGCGAAGGCGACTTTCTGGTCTGTAACTGACACTGAGGCGCGAAAGCGTGGGGAGCAAACAGGATTAGATACCCTGGTAGTCCACGCCGTAAACGATGAGTGCTAAGTGTTAGAGGGTTTCCGCCCTTTAGTGCTGCAGCTAACGCATTAAGCACTCCGCCTGGGGAGTACGGCCGCAAGGCTGAAACTCAAAGGAATTGACGGGGGCCCGCACAAGCGGTGGAGCATGTGGTTTAATTCGAAGCAACGCGAAGAACCTTACCAGGTCTTGACATCCTCTGACAACCCTAGAGATAGGGCTTTCCCCTTCGGGGGACAGAGTGACAGGTGGTGCATGGTTGTCGTCAGCTCGTGTCGTGAGATGTTGGGTTAAGTCCCGCAACGAGCGCAACCCTTGATCTTAGTTGCCAGCATTCAGTTGGGCACTCTAAGGTGACTGCCGGTGACAAACCGGAGGAAGGTGGGGATGACGTCAAATCATCATGCCCCTTATGACCTGGGCTACACACGTGCTACAATGGATGGTACAAAGGGCTGCAAACCTGCGAAGGTAAGCGAATCCCATAAAGCCATTCTCAGTTCGGATTGCAGGCTGCAACTCGCCTGCAGAAGCCGGATCGCTAGT-3’ |
